# Expression levels of inositol phosphorylceramide synthase modulate plant responses to biotic and abiotic stress in *Arabidopsis thaliana*

**DOI:** 10.1371/journal.pone.0217087

**Published:** 2019-05-23

**Authors:** Elizabeth C. Pinneh, Rhea Stoppel, Heather Knight, Marc R. Knight, Patrick G. Steel, Paul W. Denny

**Affiliations:** 1 Department of Biosciences, Durham University, Durham, United Kingdom; 2 Department of Chemistry, Durham University, Durham, United Kingdom; 3 Bayer AG, Crop Science Division, Industriepark Höchst, Frankfurt am Main, Germany; National Taiwan University, TAIWAN

## Abstract

This research was undertaken to investigate the global role of the plant inositol phosphorylceramide synthase (IPCS), a non-mammalian enzyme previously shown to be associated with the pathogen response. RNA-Seq analyses demonstrated that over-expression of inositol phosphorylceramide synthase isoforms *At*IPCS1, 2 or 3 in *Arabidopsis thaliana* resulted in the down-regulation of genes involved in plant response to pathogens. In addition, genes associated with the abiotic stress response to salinity, cold and drought were found to be similarly down-regulated. Detailed analyses of transgenic lines over-expressing *At*IPCS1-3 at various levels revealed that the degree of down-regulation is specifically correlated with the level of *IPCS* expression. Singular enrichment analysis of these down-regulated genes showed that *At*IPCS1-3 expression affects biological signaling pathways involved in plant response to biotic and abiotic stress. The up-regulation of genes involved in photosynthesis and lipid localization was also observed in the over-expressing lines.

## Introduction

According to UN estimates, growing at a rate of 1.1% per year, the world population is set to reach 9.8 billion by 2050 [1], which would require a 70% increase in food production [2]. A finite amount of arable land, coupled with the detrimental effects of climate change on crop yields, mean that strategies other than intensification will need to be employed to increase production. One that is being adopted, in combination with intensification, is the use of biotechnology to produce genetically modified crops with enhanced yields. The ability to make plants that are more tolerant to biotic and abiotic stress is predicated on identifying molecular targets which modulate plant stress responses.

One such target of interest is the non-mammalian plant enzyme inositol phosphorylceramide synthase (IPCS) which catalyses a key step in sphingolipid biosynthesis. Complex sphingolipids can be grouped into two main classes in plants: glycosylceramides and derivatives of inositol phosphorylceramide (IPC) [3]. IPCS is central to synthesis of the latter, catalyzing the transfer of phosphoinositol from phosphatidylinositol to ceramide to form IPC [4]. Ceramide is the base unit of complex sphingolipids and is composed of a long chain base (LCB) and a fatty acid (FA) component [5]. The structural diversity of complex sphingolipids is conferred by the FA and LCB, with variation in carbon chains (C16-26), hydroxylation and desaturation, and the addition of various saccharides/oligosaccharides attached, via a phosphoinositol group in some cases, to the primary hydroxyl of ceramide. These modifications account for the 168 sphingolipid species identified in *Arabidopsis thaliana* [6], and are involved in a plethora of biological pathways, including programmed cell death (PCD) [7], reproduction [8], senescence [9] and cold acclimation [10]. Disruption of the sphingolipid pathway has repeatedly been shown to be inextricably connected to plant defense signaling [11].

First identified in wax bean microsome [12] and later in *A*. *thaliana* [4, 13], IPCS has been shown to play a role as a negative regulator of PCD [13] and is required for reproduction and normal growth [14]. *In planta* three IPCS isoforms exist, and further characterization in *Oryza sativa* showed that the expression of all three *IPCS* isoforms was temporally altered to varying degrees in a tissue and stress specific manner [15]. For example, under cold stress *OsIPCS1* (NP_001044812) and *OsIPCS2* (NP_001055712) were up-regulated in roots and stems, but down-regulated in leaves; in contrast *OsIPCS3* (NP_001055096) was up-regulated in all tissues. Together, these results suggested that *OsIPCS1-3* have key roles in rice growth and abiotic stress responses [15]. With respect to the plant biotic stress response, T-DNA insertion mutants of *AtIPCS2* (AT2G37940) in *A*. *thaliana* showed increased levels of ceramide and phytoceramide, both well-documented inducers of PCD, and displayed necrotic lesions associated with PCD [13]. When exposed to the biotropic pathogen *Golovinomyces cichoracearum* UCSC1, these plants showed a reduction in fungal mass compared to controls [13]. *At*IPCS1 (AT3G54020) and *At*IPCS3 (AT2G29525) have not been characterized so far.

The data from both monocot *O*. *sativa* (rice) and dicot *A*. *thaliana* [13, 15] indicate that manipulation of IPCS activity, chemically or genetically, could be used to modulate biotic and abiotic plant stress responses. To explore this further, in this study, *A*. *thaliana* lines over-expressing each *IPCS* isoform were created and RNA-Seq carried out to monitor conserved changes in the transcriptome.

## Materials and methods

### Over-expression of *At*IPCS1-3 in *Arabidopsis thaliana*

PCR products of the full-length cDNA of *AtIPCS1-3* [4] were cloned into pENTR/D-TOPO using T4-ligase (ThermoFisher) and into the destination vector pK7WG2 [16] via Gateway LR Clonase (ThermoFisher) to create pK7WG2_AtIPCS1-3.

Primers:

*At*IPCS1-NotI-F:

GCGCGCGGCCGCCACAATGACGCTTTATATTCGCCGCG

*At*IPCS1-AscI-R:

GCGCGGCGCGCCTCATGTGCCATTAGTAGCATTATCAGTGTG

*At*IPCS2-NotI-F:

GCGCGCGGCCGCCACAATGACACTTTATATTCGTCGTGAATCTTCCAAG

*At*IPCS2-AscI-R:

GCGCGGCGCGCCTCACGCGCCATTCATTGTGTTATC

*At*IPCS3-NotI-F:

GCGCGCGGCCGCCACAATGCCGGTTTACGTTGATCGC

*At*IPCS3-AscI-R:

GCGCGGCGCGCCTCAATGATCATCTGCTACATTGTTCTCGTTT

*Agrobacterium tumefaciens strain* C58C1 was transformed with pK7WG2_AtIPCS1-3, transformants plated on Luria broth (100 μg/μl rifampicin and 100 μg/μl spectinomycin) and incubated for 3 days at 28°C. Col-0 wild-type *A*. *thaliana* were subsequently transformed using the floral dipping method [17].

### *Arabidopsis thaliana* growth conditions

Col-0 and *AtIPCS1*, *2* and *3* over-expressing plants were grown for 10 days on Murasige and Skoog (MS) agar before transfer to peat plugs. Growth conditions were 20 °with a 16-hour day / 8-hour night cycle.

### RNA preparation

RNA extraction was carried out on samples flask frozen in nitrogen, using the ReliaPrep™ Tissue Miniprep System (Promega) according to the manufacturer’s protocol. Following DNase (ThermoFisher) treatment, the integrity of the RNA was determined by running the samples on a 2100 Bioanalyzer (Agilent) to obtain an RNA Integrity Number (RIN) score.

### Quantification of *At*IPCS1-3 in over-expressing *Arabidopsis thaliana* transgenic lines

cDNA samples prepared as above and the Applied Biosystems 7300 Real-Time PCR System and the SYBR Green Jump-Start Taq Ready Mix were used to quantify transcript as previously described [4, 18, 19]. Gene specific primers were designed using Primer3plus (http://www.bioinformatics.nl/cgi-bin/primer3plus/primer3plus.cgi) for real-time PCR with PEX4 used as a reference gene. Primers: *At*IPCS1_F: TGCGTCCCGTAAACATTACA, *At*IPCS1_R: ACACCGTTCCCATTCAAGAG, A*t*IPCS2_F: TACCAGATCGGACTGCTGTG, *At*IPCS2_F: GTGAACTCCGTTGCTGTCAA, *At*IPCS3_F: CTGGGCCGAATTATCATTGT, *At*IPCS3_R: CCTTCGTGTGCCGTATCTTT

### RNA-Seq

Single end libraries for RNA-Seq were generated from DNase treated total RNA using TruSeq Stranded mRNA sample preparation kit according to manufacturer’s instructions (Illumina). Briefly, mRNAs were fragmented and purified for use as template for the synthesis of double stranded cDNA. End repair of the double stranded cDNA was carried out and the 3’ end adenylated. Sample specific indexing adapters were ligated to the ends of double stranded cDNA samples, amplified by PCR and then purified. Samples were normalized, pooled and then sequenced using a NextSeq 500 instrument (Illumina) to obtain 150 base pair single end reads.

#### RNA-Seq analyses

The RNA sequence data in Fastq format 11 were filtered and trimmed (sliding window 4:15 and 50 bp minimum) to remove low quality reads using Trimmomatic [20]. Reads were aligned to the *Arabidopsis* genome (*Arabidopsi*s Araport 2017) using STAR [21]. The sequence alignment files were sorted by name14 for HTSeq-count and indexed using SAMtools [22]. Files were converted to BAM files and number of reads mapped onto a gene calculated using HTSeq package [23]. Gene counts were normalized and compared sample by sample using DESeq2 [24] (Bioconductor [25]) in R [26]. Differential expression was determined using with a log_2_ fold-change output. GO term enrichment was performed for analyses of genes up- and down-regulated in both biological replicates using the agriGO analysis tool (http://bioinfo.cau.edu.cn/agriGO/analysis.php) with the default settings [27]. Gene annotations was carried out against *Arabidopsis* gene model (TAIR9) background (https://www.arabidopsis.org/). These data are freely available in GEO (https://www.ncbi.nlm.nih.gov/geo/ GEO Accession GSE129016).

### MapMan

Analyses were carried out using MapMan 3.5.1 R2 software [28]. RNA-Seq abundance data from the At2++ transgenic line were uploaded to MapMan and log_2_ fold change selected as the experimental data set for analyses. Mapping was carried out using Ath_AGI_TAIR9_Jan2010.

### Osmotic stress assay

Seedlings were grown under standard conditions on MS agar for 8 days before floating on the various concentrations of mannitol (without MS) in a sterile culture dish under the same conditions as the plate (16h day photo-period, 20°C). Photographs were taken after 6 days.

### Pathogen stress assay

Control and over-expressing transgenic lines were grown under short day conditions for 5 weeks and leaves excised then incubated on MS agar at 37°C for 72 hours following the addition of 5μl of *Erwinia amylovora* culture grown to an OD_600_ = 0.5.

### Genevestigator

Genevestigator [29] was utilised to identify available data showing the upregulation of *At*IPCS1, *At*IPCS2 or *At*IPCS3 in *Arabidopsis thaliana* seedlings exposed to different agents and conditions.

## Results

### Identification of genes down- or up-regulated on over-expression of *AtIPCS*

**Fig 1:**
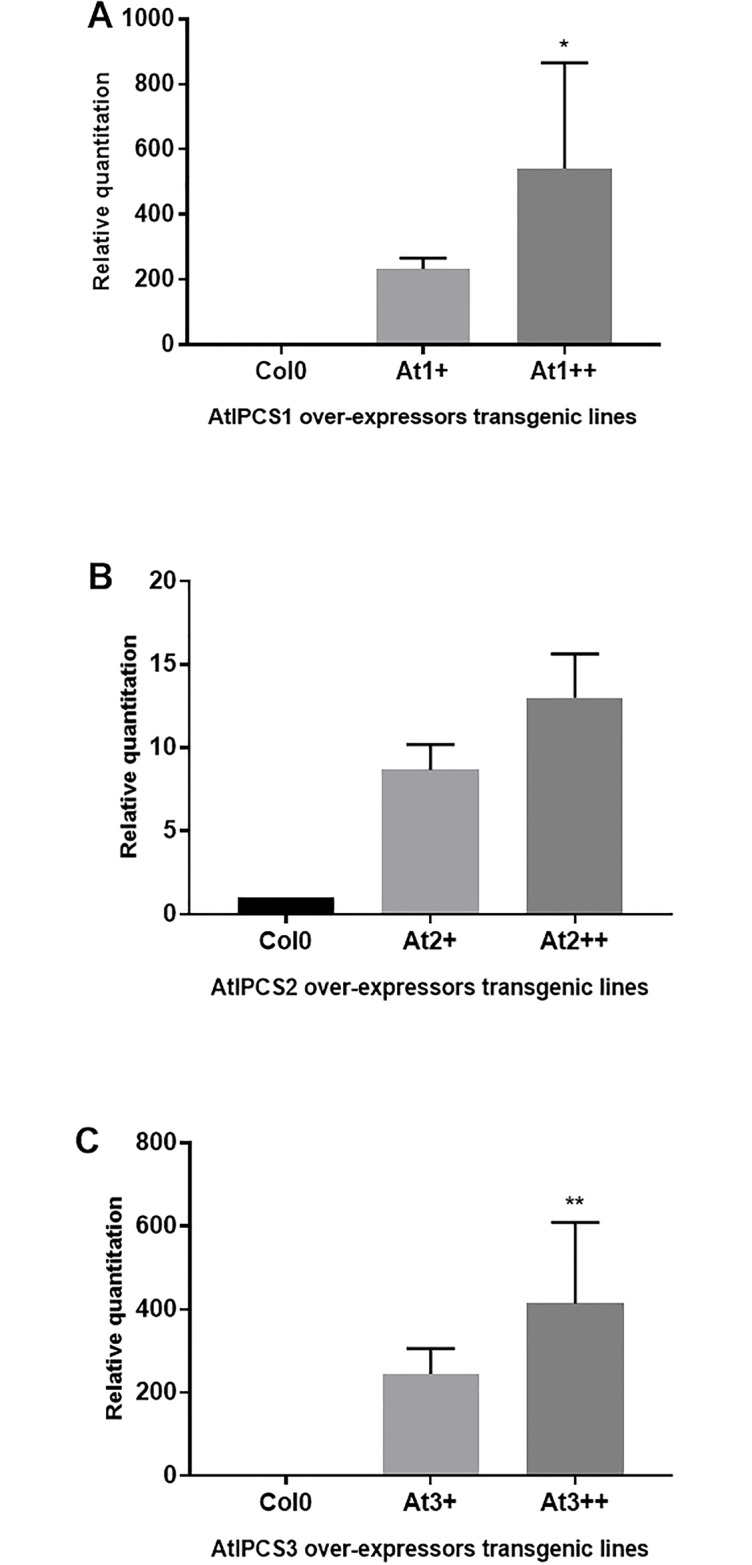
Relative quantitation of mRNA levels of (A) *At*IPCS1 (B) *At*IPCS2 (C) *At*IPCS3 in over-expressor transgenic lines compared to Col0 standardised to equate to a value of 1; qPCR was performed to measure mRNA levels in 10-day old seedlings. Relative quantitation was done after normalization using PEX4 levels; relative quantitation value is the mean of three biological replicates with standard deviation; significance of mRNA levels determined by one-way ANOVA with Turkey’s post hoc test (p < 0.05).

*A*. *thaliana* plants over-expressing the full-length cDNA of *AtIPCS1*, *AtIPCS2* and *AtIPCS3* respectively, were generated as described in Methods. For each isoform, two transgenic lines (biological replicates) over-expressing the respective *AtIPCS* were selected, one with high levels relative to wild-type Columbia-0 (Col-0) (At1-3++) and one with a lower level of over-expression (At1-3+) (Fig 1A–1C). Importantly, over-expression of one isoform did not affect the expression of the other two *At*IPCS isoforms (S1 Fig). Genome wide transcriptomic data analyses indicated that the number of expressed genes detected was very similar in all transgenic lines and in the Col-0 control (Table 1).

**Table 1:** Genes expressed in Col-0 and *AtIPCS1-3* over-expressing transgenic lines. *A*. *thaliana* Col-0 parent; At1, 2 or 3 lines over-expressing *AtIPCS1*, *2 or 3* (2 lines of each); Rep—experimental replicates (3); FPKM—Fragments Per Kilobase Million.

Genotype	Rep	Expressed genes (FPKM)	Average	Non-expressed genes (FPKM)	Average
**Col-0**	1	22740	22749	2524	2515
**Col-0**	2	22700		2564	
**Col-0**	3	22807		2457	
**At1+**	1	22410	22518	2854	2746
**At1+**	2	22503		2761	
**At1+**	3	22640		2624	
**At1++**	1	22914	22805	2350	2459
**At1++**	2	22823		2441	
**At1++**	3	22677		2587	
**At2+**	1	22643	22631	2621	2633
**At2+**	2	22546		2718	
**At2+**	3	22705		2559	
**At2++**	1	22520	22574	2744	2690
**At2++**	2	22573		2691	
**At2++**	3	22628		2636	
**At3+**	1	22657	22595	2607	2669
**At3+**	2	22593		2671	
**At3+**	3	22535		2729	
**At3++**	1	22708	22731	2556	2533
**At3++**	2	22708		2556	
**At3++**	3	22777		2487	

**Fig 2:**
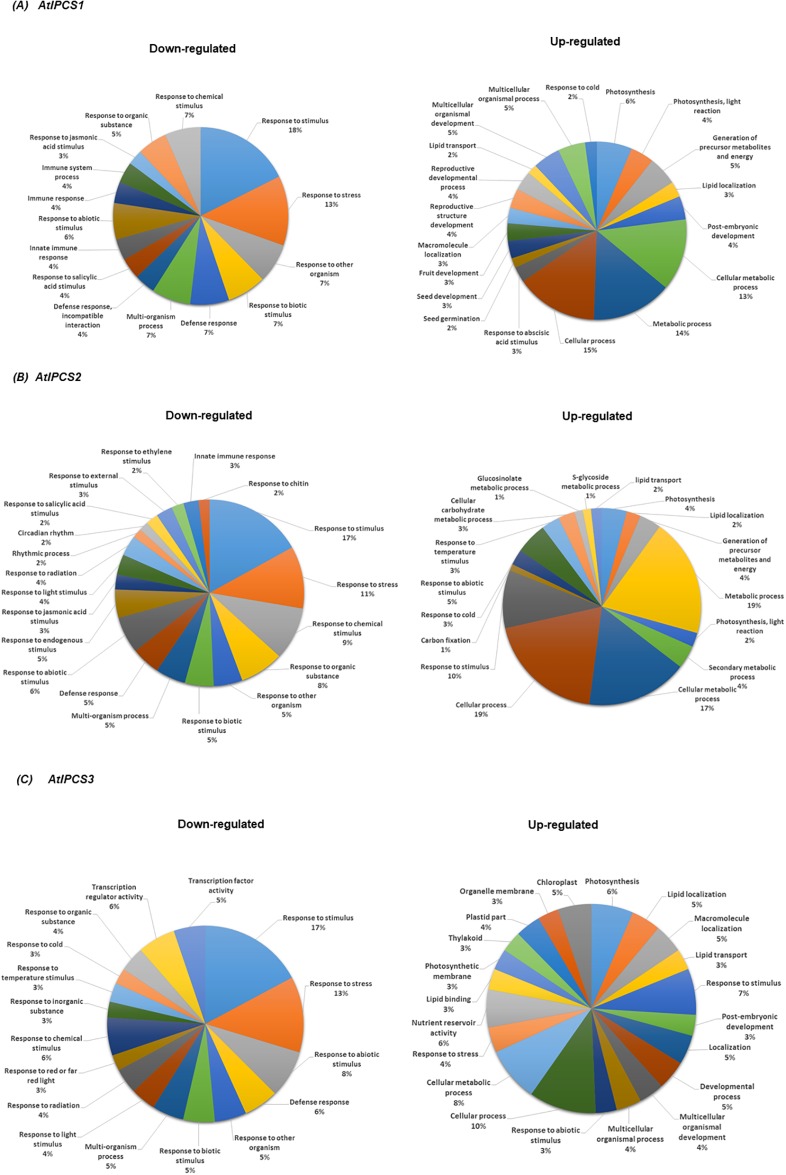
Pie chart of biological processes enriched for genes down- and up-regulated in response to the over-expression of (A) *AtIPCS1*, (B) *AtIPCS2* and (C) *AtIPCS3* isoforms when compared to wild type Col0.

**Fig 3:**
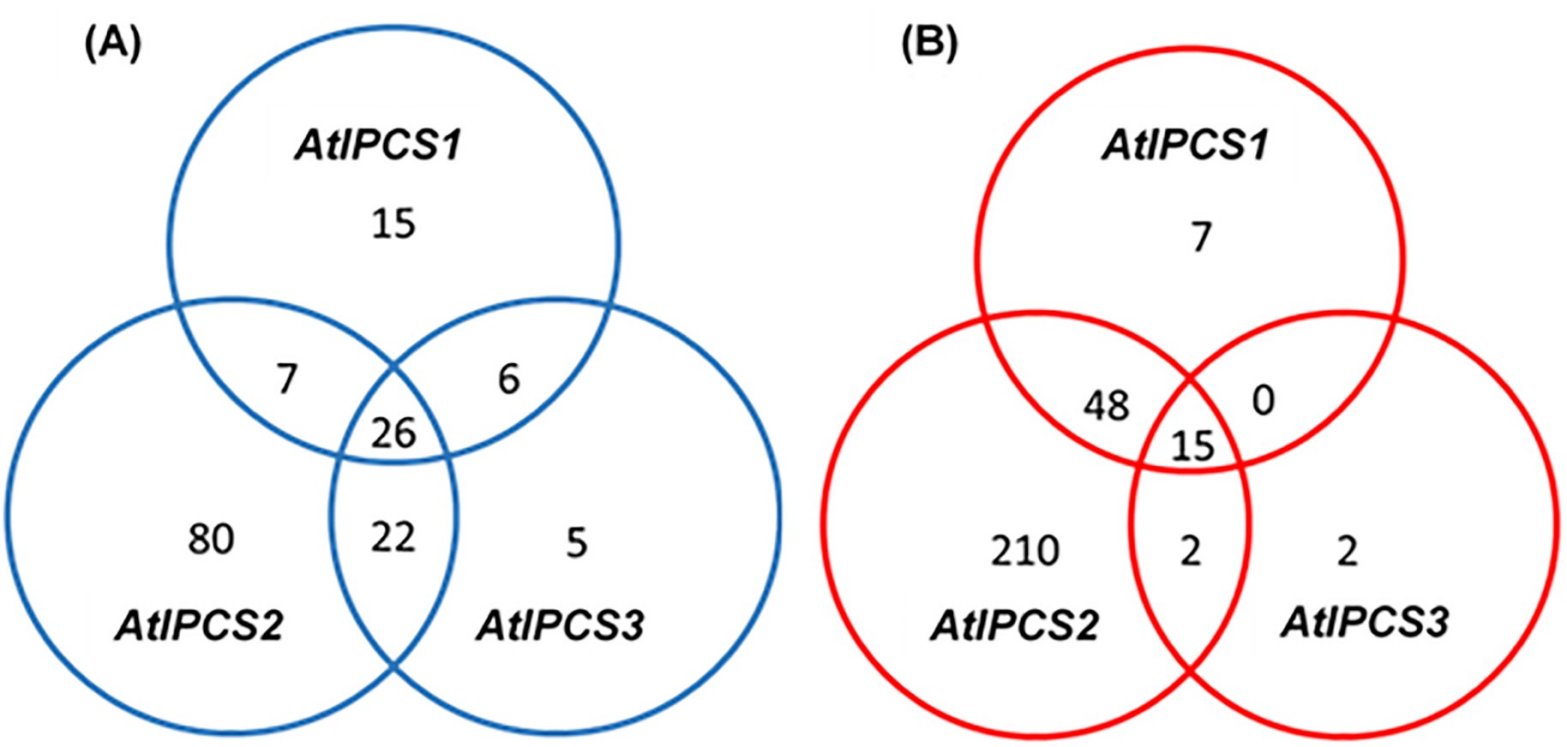
Venn diagrams of number of genes that were down-regulated (A) or up-regulated (B) in response to *AtIPCS1*, *AtIPCS2 and AtIPCS3* overexpression. Log_2_ fold change relative to wild type Col-0.

Genes that were identified as differentially expressed in both biological replicates, compared to Col0, were carried forward for further analyses. Function enrichment of the down-regulated genes using agriGO (http://bioinfo.cau.edu.cn/agriGO/index.php) revealed a significant enrichment of genes (Fisher exact test, two-tailed, p-value < 0.001) under the GO terms *response to stimulus* (p = 3.50^E-18^) and *response to stress (p =* 1.50^E-14^ *)*, whilst a significant amount of the up-regulated genes fell under the GO terms *cellular process* (p = 2.90^E-07^) and *cellular metabolic process* (p = 4.10^E-08^) (Fig 2). Those genes identified under GO term *response to stimulus* were the same genes as identified under *response to stress* and *response to abiotic stress*. Subsequently, to add specificity, focus was placed upon the latter two GO terms. *AtIPCS2* over-expression led to the down-regulation of 135 genes, considerably more than *AtIPCS1* (54) and *AtIPCS3* (59) (Fig 3A). Of these, 26 genes were down-regulated in all lines. With respect to up-regulated genes, again most (275) were in response to *AtIPCS2* over-expression, with 70 and 19 in *AtIPCS1* and *AtIPCS3* over-expressers respectively; 15 genes were found to be up-regulated in all lines (Fig 3B).

### Analyses of genes identified as responding negatively to *AtIPCS* over-expression

Global analyses of the down-regulated genes in response to *AtIPCS1* over-expression revealed significant enrichment under the GO term GO:0006950, *response to stress* (p = 1.50^E-14^), 42.6% (23/54) when they represent only 6.14% of the *Arabidopsis* transcriptome (S1 Table). Similarly, on *AtIPCS2* over-expression down-regulated genes were enriched under this term, 34.1% (46/135; p = 2.20^E-22^) (S2 Table); and on *AtIPCS3* over-expression 40.7% (24/59; p = 2.40^E-14^). Other biological processes showing a significant enrichment of genes down-regulated in these transgenic lines, included those under GO terms: GO:0042221 (*response to chemical stimulus*); GO:0010033 (*response to organic substances*), GO:0051707 (*response to other organisms*); GO:0009607 (*response to biotic stimulus*); GO:0009628 (*response to abiotic stimulus*); and GO:0006952 (*defense response*) (S1–S3 Tables).

Of particular interest were genes that showed a dose-dependent decrease in expression associated with increased *AtIPCS* expression. Genes (GO:0006950, *response to stress*) whose decrease in expression was negatively correlated (log_2_ change ≥2) with higher *AtIPCS1* levels (At1++ versus At1+], Fig 1A and Table 2) are: TYROSINE AMINOTRANSFERASE 3 (TAT3; AT2G24850); PLANT DEFENSIN 1.2B (PDF1.2B; AT2G26020); the salicylic acid inducible PATHOGENESIS-RELATED GENE, PR1 (AT2G14610) and PR2 (AT3G57260); LIPID TRANSFER PROTEIN (LTP; AT4G12490); and an ETHYLENE- AND JASMONATE-RESPONSIVE PLANT DEFENSIN (AT5G44420), a well characterized component of the defense network against pathogens [30] (Table 2).

**Table 2:** Genes showing a negative correlation with *AtIPCS* expression under GO term *GO*:*0006950* (*response to stress*) compared to Col0. At1-3+ over-expressing *AtIPCS1-3*; At1-3++ higher level expressers of *AtIPCS1-3*. Fold changes are the value of three technical triplicates of a transformed line. Transcripts listed had a p-value <0.001 (Wald test, cut off p-value < 0.05) and highlighted in bold are log_2_ fold change ≥ 2.

GeneID	Gene annotation	At1+		At1++		At2+		At2++		At3+		At3++	
		log_2_fold	p-value	log_2_fold	p-value	log_2_fold	p-value	log_2_fold	p-value	log_2_fold	p-value	log_2_fold	p-value
**AT5G47220**	ERF2 (ETHYLENE RESPONSIVE ELEMENT BINDING FACTOR 2)			**-2**	1.46E-31	-1.7	4.58E-25	-1.1	7.33E-15				
**AT2G24850**	TAT3 (TYROSINE AMINOTRANSFERASE 3)	-1.8	6.29E-17	**-4**	1.33E-53	**-2.6**	3.03E-28	**-2.3**	4.60E-26	-1.8	6.10E-18	**-2.3**	1.65E-24
**AT1G16420**	MC8 (METACASPASE 8)			**-2**	1.68E-07	-1.6	3.84E-06	-1.2	0.00047	-1	0.00242862	-1.2	0.00058
**AT4G12480**	PEARLI 1; LIPID BINDING A PUTATIVE LIPID TRANSFER PROTEIN			**-5**	1.37E-159	-1.9	1.94E-31	**-5.1**	7.83E-172	-1.2	8.99E-13	**-2**	3.20E-32
**AT1G78290**	SERINE/THREONINE PROTEIN KINASE			**-2**	2.05E-47	-1.8	8.25E-47	**-2**	1.39E-54	-1	2.73E-20	-1.3	1.51E-29
**AT4G37990**	ELI3-2 (ELICITOR-ACTIVATED GENE 3–2)			**-3**	6.02E-17	**-2.3**	7.52E-12	**-2.3**	2.18E-12	-1.8	3.22E-08	-1.6	6.77E-07
**AT3G45290**	MLO3 (MILDEW RESISTANCE LOCUS O 3)			-1	7.16E-12	-1.1	5.16E-09	-1.3	7.16E-12				3.50E-08
**AT2G26020**	PDF1.2B (PLANT DEFENSIN 1.2B)	-1.9	8.15E-18	**-5**	1.44E-66	**-4.9**	1.47E-60	**-4.9**	3.31E-62	**-2.6**	3.65E-30	**-3.4**	3.34E-40
**AT2G32680**	RLP23 (RECEPTOR LIKE PROTEIN 23)			**-2**	2.69E-17	**-2.2**	1.35E-13	**-2.3**	3.16E-15				2.85E-235
**AT2G26560**	PLA2A (PHOSPHOLIPASE A 2A)			**-3**	1.57E-78	-1.4	1.57E-26	**-2.6**	1.57E-78				2.27E-14
**AT2G14610**	PR1 (PATHOGENESIS-RELATED GENE 1)	**-3.4**	1.83E-172	**-7.3**	4.08E-238	**-7.3**	1.05E-206	**-7.9**	4.60E-217	**-4.6**	1.09E-252	**-4.9**	2.85E-235
**AT2G46830**	CCA1 (CIRCADIAN CLOCK ASSOCIATED 1)			**-2**	9.82E-14	**-2**	3.12E-12	-1.1	1.04E-05				5.91E-13
**AT1G06160**	ORA59 (OCTADECANOID-RESPONSIVE ARABIDOPSIS AP2/ERF 59)	-1	1.14E-10	-1.4	9.92E-17	**-2.5**	9.89E-37	-1.8	1.67E-24				2.31E-20
**AT3G49620**	DIN11 (DARK INDUCIBLE 11)			**-4**	1.16E-143	**-3.2**	7.04E-106	**-4.1**	5.37E-147				3.16E-94
**AT1G06040**	STO (SALT TOLERANCE)	-1.1	2.13E-28	-1.5	1.78E-49	-1.3	2.23E-38	-1.1	8.10E-27	-1	6.04E-26	-1.5	2.41E-48
**AT1G48000**	MYB112 (MYB DOMAIN PROTEIN 112)			-1	8.79E-05	**-2**	1.97E-10	-1.2	5.54E-05	-1.2	6.45E-05	-1.7	3.23E-08
**AT2G14560**	LURP1 (LATE UPREGULATED IN RESPONSE TO HYALOPERONOSPORA PARASITICA)	**-2.7**	5.62E-112	**-3.4**	1.13E-162	**-4.1**	5.67E-173	**-3.3**	1.62E-149	**-3.2**	9.88E-155	-1.7	5.56E-82
**AT1G22770**	GI (GIGANTEA)			**-2**	2.40E-75	**-2.4**	6.55E-94	**-2.3**	8.70E-91	-1.3	4.07E-33	-1.7	1.27E-94
**AT5G10140**	FLC (FLOWERING LOCUS C)	-1.2	2.18E-09	-1.5	4.16E-14	-1.4	5.23E-11	-1.3	4.55E-10	-1.1	8.80E-12	-1.7	3.25E-08
**AT3G1550**	ANAC055 (ARABIDOPSIS NAC DOMAIN CONTAINING PROTEIN 55)			**-2**	4.16E-28	-1.3	2.83E-20	-1.5	4.16E-28				
**AT1G76930**	EXT4 (EXTENSIN 4)			**-3**	1.47E-142	-1.2	1.21E-28	**-3.1**	3.77E-175				
**AT5G44420**	AN ETHYLENE- AND JASMONATE-RESPONSIVE PLANT DEFENSIN	**-2.7**	1.49E-54	**-6.1**	3.17E-118	**-6**	3.77E-111	**-6.1**	3.17E-118	**-2.7**	1.34E-55	**-3.6**	7.78E-78
**AT2G23680**	STRESS-RESPONSIVE PROTEIN			-1	3.11E-14	-1.1	3.58E-14	-1.3	1.09E-18				
**AT1G61120**	TPS04 (TERPENE SYNTHASE 04)			**-3**	1.57E-22	**-2.6**	2.43E-15	-1.7	5.55E-08	-1.6	4.61E-07	-1.8	6.72E-09
**AT1G75040**	PR5 (PATHOGENESIS-RELATED GENE 5)	-1.4	3.27E-22	-1.7	2.13E-33	-1.5	1.00E-25	-1.8	5.50E-34				
**AT1G52890**	ANAC019 (ARABIDOPSIS NAC DOMAIN CONTAINING PROTEIN 19)			**-2**	4.84E-09	-1.2	2.90E-05	-1.5	5.04E-07				
**AT3G57260**	PR2 (PATHOGENESIS-RELATED GENE 2)	-1.9	1.17E-30	**-5.3**	4.64E-102	-**4.8**	2.99E-86	-**4.4**	4.08E-87	**-2**	2.97E-36	**-2.2**	8.40E-38
**AT1G18330**	EPR1 (EARLY-PHYTOCHROME-RESPONSIVE1)			**-2**	3.87E-53	-1.6	3.85E-29	-1.9	4.62E-40	-1.3	1.98E-23	-1.6	6.68E-30
**AT1G78410**	VQ MOTIF-CONTAINING PROTEIN			-1	2.59E-05	-1.8	9.42E-08	-1.6	1.24E-06	-1.1	0.00072986	-1.2	0.00016391
**AT5G19880**	PEROXIDASE					-1.4	0.00010488	-1	0.00418				
**AT2G18050**	HIS1-3 (HISTONE H1-3)			-1	8.40E-31	**-2.5**	2.90E-96	-1.7	3.90E-55				
**AT3G04720**	PR4 (PATHOGENESIS-RELATED 4)			-1	8.36E-46	-1.6	7.55E-69	-1.5	1.20E-55				
**AT2G43510**	TI1; SERINE-TYPE ENDOPEPTIDASE INHIBITOR			**-4**	2.98E-227	**-2.5**	6.34E-132	**-3.9**	2.98E-227	-1.1	2.90E-37	-1.8	9.93E-77
**AT3G45860**	RECEPTOR-LIKE PROTEIN KINASE	-1.5	6.64E-09	**-2.9**	8.20E-25	**-2.9**	3.03E-22	**-3**	8.20E-25	-1.5	4.12E-09	-1.7	4.81E-10
**AT3G51660**	MACROPHAGE MIGRATION INHIBITORY FACTOR FAMILY PROTEIN			**-2**	6.63E-15	-1.2	1.40E-16	-1.1	6.63E-15				
**AT1G71030**	MYBL2 (ARABIDOPSIS MYB-LIKE 2)			**-2**	4.20E-88	-1.2	1.04E-29	-1.7	4.26E-55				
**AT1G57630**	DISEASE RESISTANCE PROTEIN (TIR CLASS)			**3.1**	1.80E-25	-1.5	8.34E-09	**-2.5**	4.73E-18				
**AT5G59780**	MYB59 (MYB DOMAIN PROTEIN 59)			**-2**	1.15E-47	-1.2	6.92E-29	-1.7	9.77E-56				
**AT4G12490**	LIPID TRANSFER PROTEIN (LTP)	-1.3	7.93E-05	**-4.6**	1.76E-45	**-2.8**	8.38E-18	**-4.7**	1.76E-45	-1.9	1.32E-08	**-2.6**	1.46E-15
**AT4G23210**	PROTEIN KINASE FAMILY PROTEIN ENCODES A CYSTEINE-RICH RECEPTOR-LIKE KINASE (CRK13)			**-3**	1.31E-52	-1.7	8.20E-24	**-3**	1.31E-52				
**AT1G09080**	ATP BINDING BIP3	-1.1	7.65E-06	**-2.5**	6.79E-21	-1.6	4.53E-10	-1.6	6.04E-11				
**AT4G02520**	GSTF2 (GLUTATHIONE S-TRANSFERASE PHI 2)			**-2**		-1.2	2.82E-06	**-2.9**	4.44E-28	-1.4	3.33E-08	-1.6	3.75E-10
**AT2G25000**	WRKY60					-1.2	7.64E-11	-1.4	9.79E-15				
**AT1G08320**	TGA9			-1.1	1.21E-22								
**AT3G52430**	PAD4			-1.0	1.59E-15								
**AT1G80840**	WRKY40			-1.7	5.02E-23								
**AT4G31800**	WRKY18			-1.0	1.95E-20								
**AT1G01560**	MPK11			**-2.0**	5.24E-11			-1.5	4.20E-07				
**AT1G73805**	SARD1			-1.5	3.18E-29	-1.3	5.55E-20	-1.8	1.56E-35	-1.3	6.19E-23		
**AT4G21534**	SPHINGOSINE KINASE 2							-1.2	3.12E-21				
**AT3G23250**	MYB15			-1.9	5.76E-16			**-2.4**	1.09E-20			-1.4	4.96E-10
**AT4G25470**	DREB1C							-1.3	3.23E-04	-1.1	2.06E-03	-1.1	1.21E-03
**AT4G06746**	RAP2.9					-1.1	3.27E-13						
**AT4G25470**	DREB1B			-1.1	0.0014182							-1.0	3.36E-03

Interestingly, the dose response effects noted was largely specific for the *AtIPCS1* isoform (Table 2). However, it is immediately clear that the plant defense system is more sensitive to increased *At*IPCS2 expression (At2+ and At2++), despite the increase being relatively small (up to 10-fold) compared to *At*IPCS1 and 3 (up to 390 and 440-fold respectively) (Fig 1A–1C and Table 2). The overall impact of *AtIPCS2* over-expression may be due to an increases in what represents the most abundant *AtIPCS* transcript (approximately 100-fold *AtIPCS1*) in all tissues of wild type *A*. *thaliana* [4]. Interestingly, there is no major dose response apparent on an increase in the over-expression of *AtIPCS3* (At3++ versus At3+, Table 2), despite an increase in *AtIPCS3* transcript similar to that observed for *AtIPCS1* (Fig 1C). However, transcripts that were down-regulated ≥2 log_2_ in *AtIPCS3* (either line) and were also suppressed on *AtIPCS1* or *AtIPCS2* over-expression include: TAT3; lipid binding A putative lipid transfer protein (PEARLI 1; AT4G12480); *PDF1*.*2B; PR1* and *PR2*; LATE UP-REGULATED IN RESPONSE TO *HYALOPERONOSPORA PARASITICA* (LURP1; AT2G14560); GIGANTEA (GI; AT1G22770); ETHYLENE- AND JASMONATE-RESPONSIVE ELEMENT PLANT DEFENSIN. Of the genes down-regulated with a log_2_ fold change ≥2 in response to *AtIPCS2* over-expression a large number were specific: ELICITOR-ACTIVATED GENE 3–2 (ELI3-2; AT4G37990); RECEPTOR LIKE PROTEIN 23 (RLP23; AT2G32680); PHOSPHOLIPASE A 2A (PLA2A; AT2G26560); CIRCADIAN CLOCK ASSOCIATED 1 (CCA1; AT2G46830); OCTADECANOID-RESPONSIVE *ARABIDOPSIS* AP2/ERF 59 (ORA59; AT1G06160); DARK INDUCIBLE 11 (DIN11; AT3G49620); EXTENSIN 4 (EXT4; AT1G76930); TERPENE SYNTHASE 04 (TPS04; AT1G61120); HISTONE H1-3 (HIS1-3;AT2G18050); SERINE-TYPE ENDOPEPTIDASE INHIBITOR (TI1; AT2G43510); GLUTATHIONE S-TRANSFERASE PHI 2 (GSTF2; AT4G02520). From these analyses, it is clear that *At*IPCS2 expression affects a wider network of genes (including some of those involved in plant defence responses) compared with *At*IPCS1 and *At*IPCS3 transgenic lines, perhaps due to its higher expression pattern in all tissues of *Arabidopsis* [4].

**Fig 4:**
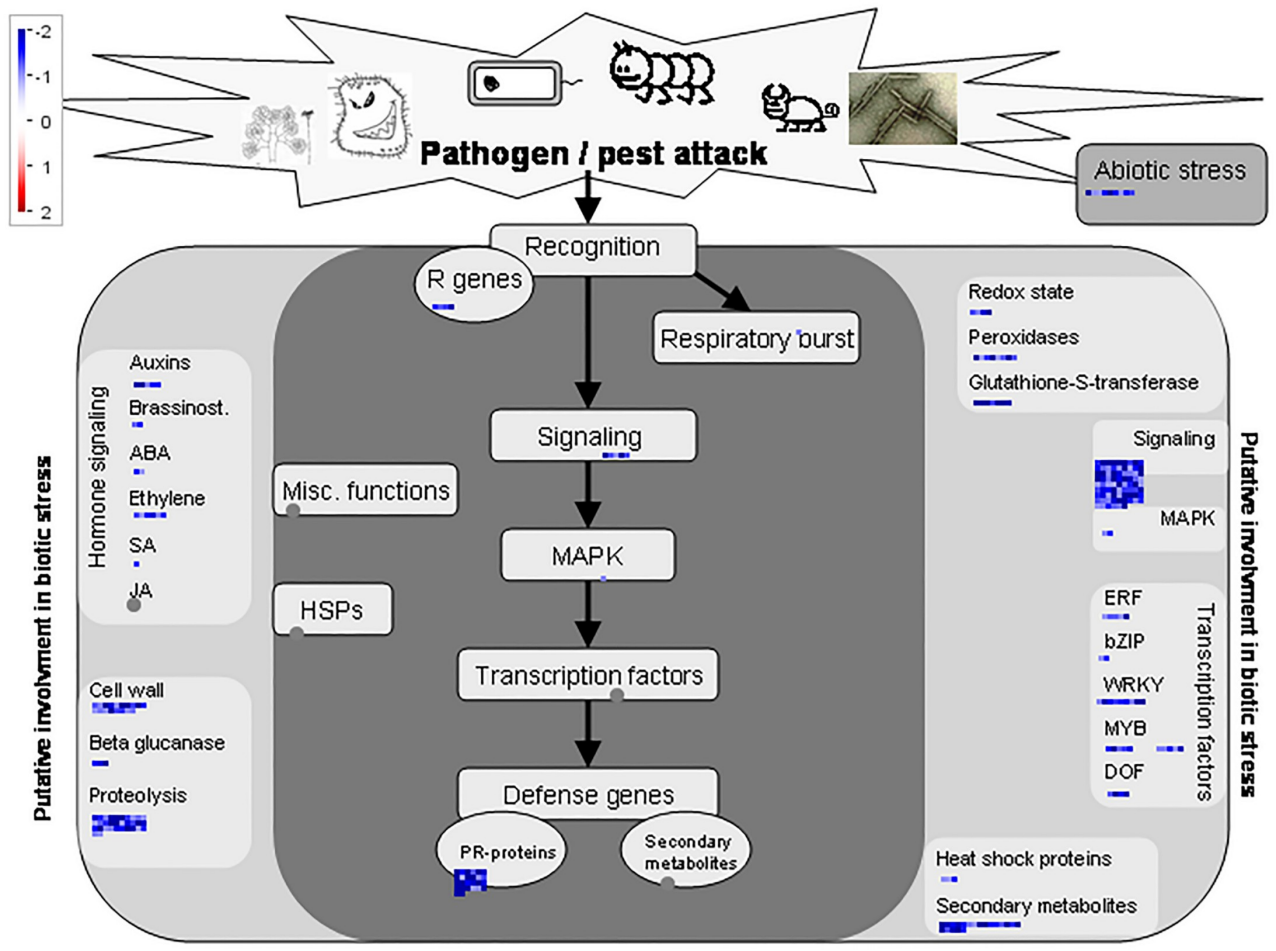
Schematic, generated in MapMan, of genes identified as down-regulated in the At2++ over-expression line and enriched under plant response to biotic stress. Log_2_ fold changes in gene expression are indicated by the colour scale. Abbreviations: Resistance (R) genes, salicylic acid (SA), jasmonic acid (JA), ethylene resposnse facotor (ERF), abscisic acid (ABA), DNA binding one zinc finger (DOF), heat-shock protein (HSP) and pathogenesis-related (PR) proteins.

The effects of over-expression (At2++) were further analysed and visualised using MapMan (https://mapman.gabipd.org). These analyses illustrated multiple negative effects on metabolism (S2 Fig), however the clearest correlation of significantly down-regulated genes (log2-fold change) in At2++ was with the plant response to biotic stress: PR proteins, peroxidases, R genes, B-glucanases, heat shock proteins, transcriptional factors and signalling proteins involved in the response to pathogens (Fig 4). Furthermore, there is a remodelling of plant architecture as seen in the down-regulation of genes involved in cell wall modifications in response to pathogen attack (Fig 4).

The 26 genes down-regulated in response to the over-expression of *AtIPCS1*, *2* and *3* are primarily involved in the plant biotic stress response to pathogens (Table 3), including BDA1 a well characterised transmembrane protein found to be necessary in the regulation and augmentation of the plant response to pathogens [31]; and AED1 [32] an aspartyl protease implicated in *Arabidopsis* systemic acquired resistance. This conserved down-regulation of genes involved in the plant response to pathogens further stresses the functional importance of *At*IPCS as a non-discriminate and far-reaching negative regulator of the response to biotic stress.

**Table 3:** Genes down-regulated in all three *AtIPCS* over-expression lines compared to Col0. At1-3+ over-expressing *AtIPCS1-3*; At1-3++ higher level expressors of *AtIPCS1-3*. Fold changes are the value of three technical triplicates of a transformed line. Transcripts listed had a p-value <0.001 (Wald test, cut off p-value < 0.05).

Gene ID	Gene annotation	At1+		At1 ++		At2+		At2++		At3+		At3++	
		log_2_ fold change	p-value	log_2_ fold change	p-value	log_2_ fold change	p-value	log_2_ fold change	p-value	log_2_ fold change	p-value	log_2_ fold change	p-value
**AT2G26020**	PDF1.2B (PLANT DEFENSIN 1.2B)	-1.9	8.15E-18	-5	1.44E-66	-4.9	1.47E-60	-4.9	3.31E-62	-2.6	3.65E-30	-3.4	3.34E-40
**AT2G24850**	TAT3 (TYROSINE AMINOTRANSFERASE 3)	-1.8	6.29E-17	-4	1.33E-53	-2.6	3.03E-28	-2.3	4.60E-26	-1.8	6.10E-18	-2.3	1.65E-24
**AT2G14610**	PR1 (PATHOGENESIS-RELATED GENE 1)	-3.4	1.83E-172	-7.3	4.08E-238	-7.3	1.05E-206	-7.9	4.60E-217	-4.6	1.09E-252	-4.9	2.85E-235
**AT1G06040**	STO (SALT TOLERANCE)	-1.1	2.13E-28	-1.5	1.78E-49	-1.3	2.23E-38	-1.1	8.10E-27	-1	6.04E-26	-1.5	2.41E-48
**AT2G14560**	LURP1 (LATE UPREGULATED IN RESPONSE TO HYALOPERONOSPORA PARASITICA)	-2.7	5.62E-112	-3.4	1.13E-162	-4.1	5.67E-173	-3.3	1.62E-149	-3.2	9.88E-155	-2.2	5.56E-82
**AT5G10140**	FLOWERING LOCUS C	-1.2	2.18E-09	-1.5	4.16E-14	-1.4	5.23E-11	-1.3	4.55E-10	-1.1	8.80E-12	-1.4	3.25E-08
**AT5G44420**	AN ETHYLENE- AND JASMONATE-RESPONSIVE PLANT DEFENSIN	-2.7	1.49E-54	-6.1	2.23E-122	-6	3.77E-111	-6.1	3.17E-118	-2.7	1.34E-55	-3.6	7.78E-78
**AT3G57260**	PR2 (PATHOGENESIS-RELATED GENE 2)	-1.9	1.17E-30	-5.3	4.64E-102	-4.8	2.99E-86	-4.4	4.08E-87	-2	2.97E-36	-2.2	8.40E-38
**AT3G45860**	RECEPTOR-LIKE PROTEIN KINASE	-1.5	6.64E-09	-2.9	3.18E-24	-2.9	3.03E-22	-3	8.20E-25	-1.5	4.12E-09	-1.7	4.81E-10
**AT4G12490**	LIPID TRANSFER PROTEIN (LTP)	-1.3	7.93E-05	-4.6	4.17E-43	-2.8	8.38E-18	-4.7	1.76E-45	-1.9	1.32E-08	-2.6	1.46E-15
**AT1G13470**	UNCHARACTERIZED	-2.1	2.12E-11	-3.7	3.73E-29	-3.3	2.67E-23	-2.8	2.70E-18	-2.1	6.88E-12	-2.4	3.04E-14
**AT1G33960**	AVRRPT2-INDUCED GENE 1	-1.5	6.39E-23	-5.4	1.23E-127	-5.7	9.38E-116	-5.2	1.80E-119	-2.2	7.45E-48	-3.3	6.79E-81
**AT3G47480**	CALCIUM-BINDING EF-HAND FAMILY PROTEIN	-1.3	3.75E-11	-3.0	3.27E-39	-2.8	4.26E-33	-3.3	6.57E-42	-1.6	4.36E-16	-2.2	5.55E-24
**AT4G03450**	ANKYRIN REPEAT FAMILY PROTEIN	-2.0	3.19E-10	-3.4	9.96E-24	-3.1	1.96E-20	-3.2	1.03E-21	-2.3	8.75E-13	-2.1	1.90E-10
**AT4G23150**	CYSTEIN-RICH RLK	-1.7	5.61E-07	-3.2	1.05E-20	-2.9	5.61E-18	-3.1	5.53E-20	-2.1	1.08E-09	-2.5	2.97E-13
**AT5G52760**	COPPER TRANSPORT PROTEIN FAMILY	-1.2	4.82E-05	-2.6	2.28E-17	-2.4	3.65E-14	-2.7	1.53E-17	-1.4	5.87E-07	-2.2	2.08E-12
**AT5G54610**	BDA1	-2.9	1.16E-25	-4.2	8.48E-45	-4.0	6.73E-39	-3.7	2.34E-36	-2.9	1.54E-26	-2.2	4.99E-16
**AT5G10760**	AED1	-1.7	2.88E-39	-4.6	1.85E-126	-4.2	6.95E-105	-4.1	1.41E-112	-2.1	1.09E-55	-1.9	6.10E-45
**AT5G60900**	RLK1	-1.1	9.02E-06	-2.9	1.86E-24	-2.3	1.51E-16	-2.8	2.43E-22	-1.3	1.25E-07	-1.5	2.05E-09
**AT2G18660**	EGC2	-1.6	2.23E-11	-3.5	8.48E-35	-3.9	7.62E-38	-3.5	1.52E-33	-2.3	8.03E-20	-2.6	6.45E-22
**AT2G26400**	ARD1	-2.0	2.90E-12	-2.2	6.94E-15	-2.9	2.32E-20	-2.3	1.82E-15	-2.4	2.33E-16	-3.1	9.05E-23
**AT2G29120**	GLR2.7	-1.2	6.10E-09	-2.3	6.40E-25	-1.8	1.01E-15	-2.0	9.39E-20	-1.0	1.54E-07	-1.4	3.64E-11
**AT3G28510**	AAA-ATPase	-2.1	2.93E-23	-1.4	2.97E-13	-2.9	1.42E-32	-1.8	1.70E-17	-1.5	2.91E-14	-2.2	6.87E-23
**AT1G52400**	BGLU18	-1.4	1.35E-49	-1.9	3.86E-91	-1.8	1.92E-85	-1.0	2.44E-28	-1.1	2.69E-34	-1.0	5.51E-28
**AT1G32960**	SBT3.3	-1.4	5.56E-06	-3.4	1.61E-25	-3.5	3.54E-26	-3.2	2.01E-22	-2.0	6.04E-11	-2.0	5.74E-10
**AT3G17609**	HYH	-1.7	1.92E-14	-1.5	3.92E-12	-1.6	8.78E-12	-1.5	4.75E-11	-1.5	1.75E-12	-1.7	1.87E-13

Genes involved in the plant response to abiotic stress are also highlighted as down-regulated in Fig 4, although they did not map to specific pathways. Notably, the sphingolipid biosynthetic pathway itself has been linked to the abiotic stress response. Only one gene in this pathway, sphingosine kinase (AT4G21534), had significantly altered expression (down-regulated) in At2++ (see data deposited in GEO, Accession GSE129016). The protein product of this gene phosphorylates sphingosine (to S1P) and phytosphingosine (to phytoS1P) in plants [33], and increased levels of S1P and abscisic acid dependent stomatal closure have been reported in response to drought [34]. Knock-down of sphingosine kinase expression significantly decreased sensitivity to abscisic acid induced stomatal closure compared to Col0 [35], indicating that At2++ with reduced sphingosine kinase may be more sensitive to drought. One gene showed a negative correlation under GO term *GO*:*009819* (*drought recovery*), a serine/theronine kinase (AT1G78290), a member of the SNF1-related protein kinase (SnRK2) family whose activity is activated by osmotic stress and dehydration [36]. Similarly, DREB genes have been implicated in the plant response to abiotic stress, including osmotic stresses such as high salinity and drought [37], and DREB1B and DREB1C were down-regulated in all the higher level over-expressors (At1-3++; Table 2). Together, these data place *At*IPCS at the heart of the abiotic, as well as biotic, stress response.

Overall, these data show a complex picture of the modulation of the plant stress response on over-expression of *AtIPCS* isoforms. Some changes were relatively specific for an isoform, some showed dose response effects that correlated with *At*IPCS expression levels, and all are genes modulated in response to biotic and abiotic stress.

### Analyses of genes identified as responding positively to *AtIPCS* over-expression

Analyses of genes whose expression was positively correlated with *AtIPCS1* over-expression (At1+ and At1++) revealed a significant enrichment (Fisher exact test, two tailed, p < 0.001) for those under GO term GO:0015979, *photosynthesis* (p = 9.10^E-27^) 25.7% (18/70) when they represent 0.43% of the *Arabidopsis* transcriptome (S4 Table). Similarly, for *AtIPC*2 over-expressor transgenic lines (At2+ and At2++) there was a significant enrichment (p = 1.70^E-31^) with 13.3% (30/226) of the genes associated under this GO term (S5 Table). *AtIPC*3 over-expressor lines (At3+ and At3++) also had a significant enrichment under *photosynthesis* (p = 6.00^E-18^)—34.5% (10/29). Other GO terms with significant enrichment for up-regulated genes in response to *AtIPCS1*, *2* or *3* over-expression include: GO:0010876 (*lipid localization*), GO:0006091 (*generation of precursor metabolites and energy*) and GO:0033036 (macromolecular localization) (S4–S6 Tables).

Utilising the differential over-expression in the analysed lines, the influence of *AtIPCS1-3* expression levels on gene up-regulation was analysed. A dose dependent increase in the expression of up-regulated genes under GO term GO:0015979 (*photosynthesis*) was associated with the increase in *AtIPCS1* and *2* transcript found in the transgenic lines (At1+ and At1++, and At2+ and At2++; Table 4). Those showing ≥2 log_2_ fold change dose response to both *AtIPCS1* and *AtIPCS2* are: YCF4, a PROTEIN REQUIRED FOR PHOTOSYSTEM I ASSEMBLY AND STABILITY (ATCG00520); a SUBUNIT OF THE CHLOROPLAST NAD(P)H DEHYDROGENASE COMPLEX, NDHI (ATCG01090); NADH DEHYDROGENASE ND1 NDHA (ATCG01100); PSAC SUBUNIT OF PHOTOSYSTEM I (ATCG01060); 49KDA PLASTID NAD(P)H DEHYDROGENASE SUBUNIT H PROTEIN (ATCG01110); NDHC, NADH DEHYDROGENASE D3 SUBUNIT OF THE CHLOROPLAST NAD(P)H DEHYDROGENASE COMPLEX (ATCG00440); YCF3, a PROTEIN REQUIRED FOR PHOTOSYSTEM I ASSEMBLY AND STABILITY (ATCG00360). All isoforms clearly influence the expression of the genes under this GO term and are therefore likely to influence photosynthesis itself. Over-expression of *AtIPCS2* has the broadest and largest effect, correlating again with its status as the most abundant, and perhaps most important, *AtIPCS* isoform (approximately 100-fold *AtIPCS1*) in all tissues of wild type *A*. *thaliana* [4].

**Table 4:** Genes showing a positive correlation with *AtIPCS* expression under GO term GO:0015979 (*photosynthesis*). At1-3+ over-expressing *AtIPCS1-3*; At1-3++ higher level expressers of *AtIPCS1-3*. Fold changes are the value of three technical triplicates of a transformed line. Transcripts listed had a p-value <0.001 (Wald test, cut off p-value < 0.05) and highlighted in bold log_2_ fold change ≥ 2.

GeneID	Gene annotation	At1+		At1++		At2+		At2++		At3+		At3++	
		log_2_fold	p-value	log_2_fold	p-value	log_2_fold	p-value	log_2_fold	p-value	log_2_fold	p-value	log_2_fold	p-value
**ATCG00520**	ENCODES A PROTEIN REQUIRED FOR PHOTOSYSTEM I ASSEMBLY AND STABILITY	1.3	0.000136	**2**	6.06E-09	**2.4**	3.56E-12	**3.6**	7.23E-26			**2**	1.23E-08
**ATCG01090**	ENCODES SUBUNIT OF THE CHLOROPLAST NAD(P)H DEHYDROGENASE COMPLEX NDHI	1.5	1.14E-05	**2.5**	1.60E-13	**2.7**	1.01E-15	**4**	4.89E-33			**2.2**	6.40E-11
**ATCG00540**	ENCODES CYTOCHROME F APOPROTEIN	1.1	0.00043	1.9	3.70E-09	**2.2**	1.72E-12	**3.4**	7.67E-27			1.7	3.65E-08
**ATCG01010**	CHLOROPLAST ENCODED NADH DEHYDROGENASE UNIT. NDHF					**2.2**	2.06E-10	**3.4**	7.33E-22			1.8	2.59E-07
**ATCG00280**	CHLOROPLAST GENE ENCODING A CP43 SUBUNIT OF THE PHOTOSYSTEM II REACTION CENTER	1.5	2.31E-06	1.6	1.08E-06	1.9	2.22E-09	**3**	1.05E-20			1.7	7.88E-08
**ATCG00680**	ENCODES FOR CP47, SUBUNIT OF THE PHOTOSYSTEM II REACTION CENTER	1	0.000857	1.2	0.000146	1.5	8.93E-07	**2.5**	2.18E-15			1.3	2.90E-05
**AT4G28660**	PSB28 (PHOTOSYSTEM II REACTION CENTER PSB28 PROTEIN)					1.6	6.17E-59	1.2	8.53E-37	0.5			
**ATCG01100**	NADH DEHYDROGENASE ND1 NDHA	1.6	2.92E-06	**2.6**	2.62E-15	**2.9**	1.82E-18	**4**	3.49E-33			**2.4**	9.53E-13
**AT3G04790**	RIBOSE 5-PHOSPHATE ISOMERASE-RELATED					1.8	1.32E-101	1.3	6.95E-58	0.8		1.3	1.00E-52
**ATCG00270**	PSII D2 PROTEIN PSBD	1.8	5.79E-08	1.9	4.30E-09	**2.4**	7.28E-13	**3.4**	1.13E-25			**2.1**	3.43E-10
**ATCG01060**	ENCODES THE PSAC SUBUNIT OF PHOTOSYSTEM I	1.3	5.19E-05	**2.2**	3.40E-12	**2.6**	8.05E-17	**3.6**	2.28E-30			**2.1**	1.50E-11
**AT3G55800**	SBPASE (SEDOHEPTULOSE-BISPHOSPHATASE);					1.2	1.64E-100	1	4.49E-67	0.3			
**AT4G09650**	ATPD (ATP SYNTHASE DELTA-SUBUNIT GENE);					1.5	7.39E-119	1.2	3.91E-76	0.3		1.1	3.83E-57
**ATCG00730**	A CHLOROPLAST GENE ENCODING SUBUNIT IV OF THE CYTOCHROME B6	1.3	2.93E-05	1.6	1.02E-06	1.9	4.38E-09	**2.8**	6.43E-19			1.6	3.63E-07
**ATCG01080**	NADH DEHYDROGENASE ND6 NDHG					1.9	9.92E-08	**3.1**	5.52E-19			1.5	1.37E-05
**ATCG00710**	ENCODES A 8 KD PHOSPHOPROTEIN	1	0.000866	1.2	1.80E-06	1.7	5.64E-08	**2.8**	1.16E-17			1.5	6.42E-06
**AT2G01590**	CRR3 (CHLORORESPIRATORY REDUCTION 3)					1.2	1.06E-40	1	1.79E-31				
**ATCG00580**	PSII CYTOCHROME B559	1.1	0.0003654	1.3	8.33E-05	1.8	8.75E-09	**2.4**	2.18E-13			1.3	2.94E-05
**ATCG00720**	ENCODES THE CYTOCHROME B(6) SUBUNIT OF THE CYTOCHROME B6F COMPLEX	1.1	0.0005436	1.4	5.73E-06	1.7	7.29E-08	**2.9**	4.62E-20			1.4	5.58E-06
**AT3G01440**	PSB-LIKE PROTEIN 2 (PQL2)					1.8	7.06E-107	1.5	2.18E-13	0.5	4.69E-10	1.3	2.89E-50
**AT1G14150**	PSB-LIKE PROTEIN 1 (PQL1)					1.5	3.82E-75	1.2	1.54E-44	0.5	7.91E-09	1	4.06E-35
**ATCG01110**	ENCODES THE 49KDA PLASTID NAD(P)H DEHYDROGENASE SUBUNIT H PROTEIN	**2.8**	3.73E-19	**4**	4.72E-37	**4.2**	1.35E-40	**4.7**	5.23E-52	1	0.001626	**3.6**	5.95E-30
**AT1G19150**	LHCA6; CHLOROPHYLL BINDING PSI TYPE II CHLOROPHYLL A/B-BINDING PROTEIN	-		1.2		**2**	2.50E-114	1.6	5.98E-73	0.7	1.71E-15	1.5	1.95E-66
**AT1G60950**	FED A; 2 IRON, 2 SULFUR CLUSTER BINDING					1.6	1.03E-63	1.5	1.68E-54			1.2	1.99E-33
**ATCG00300**	ENCODES PSBZ, WHICH IS A SUBUNIT OF PHOTOSYSTEM II	1.1	0.0022523	1.6	6.17E-06	1.7	8.98E-07	**3.2**	1.04E-19			1.6	2.72E-06
**ATCG00440**	ENCODES NADH DEHYDROGENASE D3 SUBUNIT OF THE CHLOROPLAST NAD(P)H DEHYDROGENASE COMPLEX NDHC	1.3	0.0001679	**2**	3.59E-09	**2.7**	6.65E-16	**3.4**	4.14E-24			1.9	1.08E-08
**ATCG00340**	ENCODES THE D1 SUBUNIT OF PHOTOSYSTEM I AND II REACTION CENTERS. PSAB	1.2	0.0001204	1.6	1.29E-06	1.8	1.35E-08	**2.9**	4.71E-19			1.6	1.30E-06
**ATCG00360**	ENCODES A PROTEIN REQUIRED FOR PHOTOSYSTEM I ASSEMBLY AND STABILITY	1.5	6.99E-06	**2.3**	5.20E-12	**2.7**	1.25E-15	**3.8**	1.03E-29			**2.1**	4.94E-10
**AT3G16250**	NDF4 (NDH-DEPENDENT CYCLIC ELECTRON FLOW 1);					1.4	1.02E-79	1.1	5.91E-52	0.4	1.70E-07		
**AT3G62410**	PROTEIN BINDING CP12-2 ENCODES A SMALL PEPTIDE FOUND IN THE CHLOROPLAST STROMA					1.8	5.62E-79	1.4	5.57E-49	0.3	0.006318	1.5	2.39E-51

**Fig 5:**
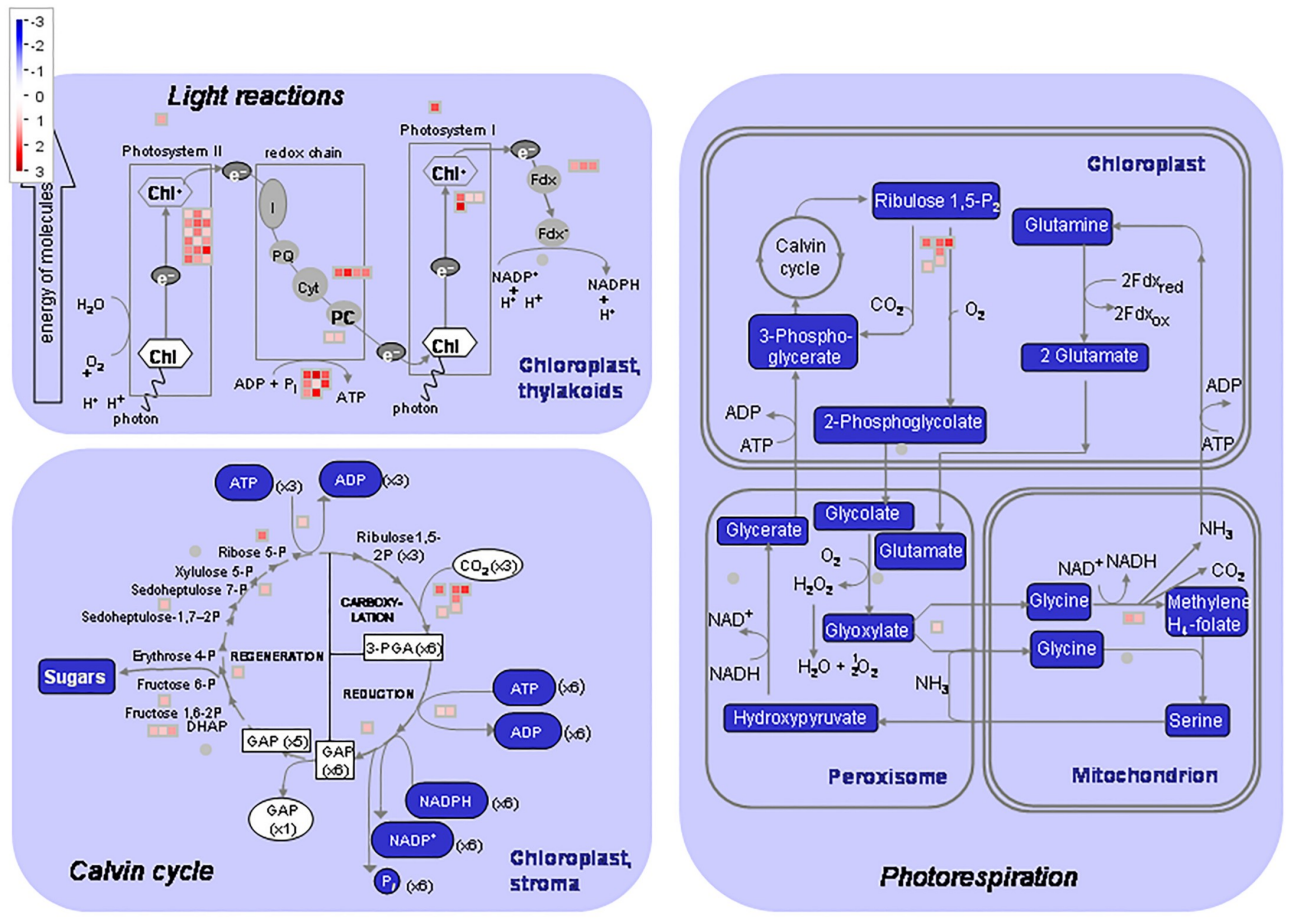
MapMan schematic of showing genes identified as upregulated in the At2++ over-expression line enriched under the plant light reaction, Calvin cycle and photorespiration. Log_2_ fold changes in gene expression are indicated by the colour scale.

The effects of over-expression (At2++) were further analysed and visualised using MapMan (https://mapman.gabipd.org). This illustrated multiple positive transcriptional effects on genes associated with metabolism (S3 Fig), a greater effect than those negatively affected (S2 Fig). Amongst those particularly influenced were genes associated with the metabolism of light reactions and flavonoids. The effects clustered under light reactions correlated with the enrichment under GO term GO:0015979, *photosynthesis* discussed above. Further analyses using MapMan showed that genes up-regulated in At2++ had high enrichment under photorespiration, the Calvin cycle and light reactions (Fig 5). This indicated that there was an associated increase in energy production and, perhaps, the rate of growth.

The MapMan analyses (S3 Fig) also indicated an upregulation of flavonoid metabolism. Flavonoids are antioxidant molecules usually produced as a result of ROS accumulation in response to abiotic and biotic stress [38]. These data indicated that over-expression of *At*IPCS2 may play a protective role in plant defense, not only as a negative regulator of plant pathogen defense genes, but also as a positive regulator of metabolites that have antioxidant properties.

After *photosynthesis*, the next best supported GO term for up-regulated genes across all *AtIPCS* isoform over-expressers was GO:0010876 (*lipid localization*; p = 8.20^E-16^, 1.80^E-26^ and 6.00^E-18^ for *AtIPCS1*, *2* and 3 respectively (S4–S6 Tables). The degree of enrichment across these genes (8/70, 17/275, and 7/19 for *AtIPCS1*, *2* and 3 respectively) was greater than that observed with GO:0015979 (*photosynthesis*); in addition, higher transcript log_2_ fold changes correlated with the rise in *AtIPCS* isoform expression levels (Table 5). Increased *AtIPCS1* expression (At1+ to At1++; 200–390 fold wild type Col-0 (Fig 1A) saw ≥2 log_2_ increase in expression of the following genes under GO:0010876: OLEOSIN 1, 2 and 4 (OLEO1, 2 and 4; AT4G25140, AT5G40420 and AT3G27660); 2S SEED STORAGE PROTEIN 1–4 (AT4G27140, AT4G27150, AT4G27160 and AT4G27170); and LIPID TRANSFER PROTEIN (LPT; AT5G54740). All of these genes were also up-regulated in response to *AtIPCS2* over-expression, including all 4 SEED STORAGE PROTEIN genes (AT4G27140, AT4G27150, AT4G27160 and AT4G27170). However, only isoforms 1, 3 and 4 increased ≥2 log_2_ in expression on further increase in *AtIPCS2* expression (At2+ to At2++; 7–9 fold wild type Col-0; Fig 1B; Table 5). In addition, LIPID TRANSFER PROTEIN 4 (LTP4; AT5G59310); LIPID BINDING PROTEIN PREDICTED TO ENCODE A PR (PATHOGENESIS-RELATED) protein (LTP6; AT3G08770); and LIPID TRANSFER PROTEIN (LPT; AT5G55410 and AT2G37870) are all up-regulated ≥2 log_2_ on over-expression of this isoform. None of these are increased ≥2 log_2_ in the higher *AtIPCS2* expressors, however LPT4 is decreased. As above, *AtIPCS2* over-expression had the broadest effect on the selected genes (GO:0010876), again presumably due to its high levels in all tissues of wildtype *A*. *thaliana* [4]. All 4 SEED STORAGE PROTEIN genes, LTP (AT5G54740) and OLEO1 and 4, are up-regulated in response to *AtIPCS3* over-expression. Furthermore, all are further up-regulated (≥2 log_2_) on increased expression (At3+ to At3++; 220–440 fold wild type Col-0; Fig 1C; Table 5). Although isoform specific effects, particularly with respect to *AtIPCS2*, were observed, over-expression of each lead to an up-regulation in expression of genes associated with GO term GO:0010876 (*lipid localization*). Analyses of the genes up-regulated in all over-expression lines demonstrated that they mainly encode seed storage proteins (Table 6), including CRUCIFERIN 2 and 3 genes (*CRU2* and *3*). *CRU2* and *3* are expressed during the later stages of embryogenesis [39], with CRU3 having a role in protein and oil storage [40]. Together, these data indicated that the increased metabolic activity, perhaps induced by *At*IPCS over-expression (S3 Fig), may result in an increase in protein and lipid storage during seed development.

**Table 5:** Genes showing a positive correlation with *AtIPCS* expression under GO term GO:0010876 (*lipid localization*). At1-3 over-expressing *AtIPCS1-3*; At1-3+ higher level expressers of *AtIPCS1-3*. Fold changes are the value of three technical triplicates of a transformed line. Transcripts listed had a p-value <0.001 (Wald test, cut off p-value < 0.05) and highlighted in bold log_2_ fold change ≥ 2.

GeneID	Gene annotation	At1+		At1++		At2+		At2++		At3+		At3++	
		log_2_fold	p-value	log_2_fold	p-value	log_2_fold	p-value	log_2_fold	p-value	log_2_fold	p-value	log_2_fold	p-value
**AT5G59310**	LTP4 (LIPID TRANSFER PROTEIN 4)					**3.7**	2.07E-28	1	0.00358				
**AT5G48490**	DIR1 LIPID TRANSFER PROTEIN					1.2	4.21E-16	1.3	4.78E-18				
**AT3G08770**	LTP6; LIPID BINDING PREDICTED TO ENCODE A PR (PATHOGENESIS-RELATED) PROTEIN					**2**	3.96E-65	1.3	4.44E-27				
**AT5G40420**	OLEO2 (OLEOSIN 2)	1.4	1.45E-07	**4.6**	3.63E-70	**2.5**	9.95E-21	**2.8**	5.74E-25				
**AT4G25140**	OLEO1 (OLEOSIN 1)	**3.5**	4.38E-45	**7.1**	2.82E-192	**4.6**	2.85E-80	**5**	1.20E-91	**2.5**	1.01E-21	**6.1**	3.11E-141
**AT4G27140**	2S SEED STORAGE PROTEIN 1	**4.2**	5.35E-65	**8**	1.03E-238	**4.3**	1.28E-68	**6.3**	1.95E-148	**3**	4.86E-32	**7.2**	6.10E-193
**AT4G27150**	2S SEED STORAGE PROTEIN 2	**3.8**	2.16E-40	**7.3**	2.16E-147	**4.7**	1.09E-61	**5.4**	1.89E-81	**2.3**	8.24E-16	**6.4**	7.03E-115
**AT4G27160**	2S SEED STORAGE PROTEIN 3	**5**	1.84E-81	**8.5**	2.51E-239	**4.2**	4.18E-58	**6.3**	1.28E-129	**3.6**	4.03E-43	**7.3**	2.59E-176
**AT5G64080**	LPT (LIPID TRANSFER PROTEIN)					1.6	1.28E-30	1.2	3.85E-18				
**AT5G05960**	LPT (LIPID TRANSFER PROTEIN)					1.7	4.62E-51	1.2	6.31E-26				
**AT3G18280**	LPT (LIPID TRANSFER PROTEIN)					1.3	7.33E-28	1.1	3.46E-19				
**AT5G54740**	LPT (LIPID TRANSFER PROTEIN)	**5.1**	3.42E-97	**8.2**	3.86E-259	**5.8**	1.57E-127	**6.3**	5.98E-153	**3.9**	4.20E-57	**7.7**	9.99E-230
**AT5G55410**	LPT (LIPID TRANSFER PROTEIN)					**2.2**	4.37E-12	**2**	1.50E-09				
**AT4G27170**	2S SEED STORAGE PROTEIN 4	**2.7**	1.03E-19	**5.7**	5.84E-88	1.8	6.48E-09	**3.9**	2.71E-39	**1.8**	6.80E-09	**4.9**	9.93E-64
**AT3G27660**	OLEO4 (OLEOSIN 4)	**2.1**	1.35E-15	**5.2**	7.26E-100	**2.8**	6.81E-27	**2.6**	5.28E-23	1.1	8.65E-05	**4**	2.28E-58
**AT2G37870**	LPT (LIPID TRANSFER PROTEIN)					**2.9**	2.88E-27	1.5	4.73E-08				
**AT3G01570**	OLE05 (OLEOSIN 5)					1.5	8.26E-13	1.3	1.92E-08				

**Table 6:** Genes up-regulated in all three *AtIPCS* over-expression lines compared to Col0. At1-3+ over-expressing *AtIPCS1-3*; At1-3++ higher level expressers of *AtIPCS1-3*. Fold changes are the value of three technical triplicates of a transformed line. Transcripts listed had a p-value <0.001 (Wald test, cut off p-value < 0.05).

GeneID	Gene annotation	At1+		At1++		At2+		At2++		At3+		At3++	
		log_2_fold	p-value	log_2_fold	p-value	log_2_fold	p-value	log_2_fold	p-value	log_2_fold	p-value	log_2_fold	p-value
AT1G03880	CRUCIFERIN 2	2.4	1.77E-17	6.9	2.47E-149	2.8	1.65E-22	4.4	2.05E-59	1.9	1.19E-10	5.5	6.22E-96
AT1G14940	POLYKETIDE CYCLASE	1.3	0.00013457	3.9	3.80E-37	2.9	1.09E-19	1.2	0.000415868	1.2	0.000363067	3.9	5.89E-37
AT1G68250	UNCHARACTERIZED	3.3	1.29E-35	5.5	4.67E-100	3.3	8.42E-35	4.7	2.00E-72	1.6	1.42E-08	4.7	1.07E-72
AT1G75830	PDF1.1	2.7	3.86E-42	6.1	4.61E-234	4.3	6.31E-116	4.1	6.34E-102	1.6	6.48E-15	5.3	1.15E-174
AT4G27150	2S SEED STORAGE PROTEIN 2	3.8	2.16E-40	7.3	2.16E-147	4.7	1.09E-61	5.4	1.89E-81	2.3	8.24E-16	6.4	7.03E-115
AT2G27380	EPR1	2.9	3.08E-31	6.0	6.18E-135	5.1	6.03E-100	2.9	4.27E-33	1.5	4.85E-09	5.6	8.36E-120
AT3G27660	OLEO4 (OLEOSIN 4)	2.1	1.35E-15	5.2	7.26E-100	2.8	6.81E-27	2.6	5.28E-23	1.1	8.65E-05	4.0	2.28E-58
AT4G25140	OLEO1 (OLEOSIN 1)	3.5	4.38E-45	7.1	2.82E-192	4.6	2.85E-80	5.0	1.20E-91	2.5	1.01E-21	6.1	3.11E-141
AT4G28520	CRU3	5.8	9.91E-131	9.6	0	6.0	5.56E-142	7.3	9.09E-207	4.2	6.51E-67	8.3	2.55E-271
AT5G44120	CRA1	5.6	2.15E-133	9.0	0	6.8	2.63E-201	6.1	5.74E-158	3.6	1.13E-55	7.9	9.89E-267
AT3G22640	PAP85	3.2	2.74E-52	5.5	5.80E-166	3.2	9.47E-51	2.6	1.55E-32	1.5	6.57E-11	4.1	4.59E-89
AT4G27160	2S SEED STORAGE PROTEIN 3	5.0	1.84E-81	8.5	2.51E-239	4.2	4.18E-58	6.3	1.28E-129	3.6	4.03E-43	7.3	2.59E-176
AT4G27140	2S SEED STORAGE PROTEIN 1	4.2	5.35E-65	8.0	1.03E-238	4.3	1.28E-68	6.3	1.95E-148	3.0	4.86E-32	7.2	6.10E-193
AT4G27170	2S SEED STORAGE PROTEIN 4	2.7	1.03E-19	5.7	5.84E-88	1.8	6.48E-09	3.9	2.71E-39	1.8	6.80E-09	4.9	9.93E-64
AT5G54740	LPT (LIPID TRANSFER PROTEIN)	5.1	3.42E-97	8.2	3.86E-259	5.8	1.57E-127	6.3	5.98E-153	3.9	4.20E-57	7.7	9.99E-230

### Phenotype of *Arabidopsis* over-expressing *AtIPCS* isoforms

**Fig 6:**
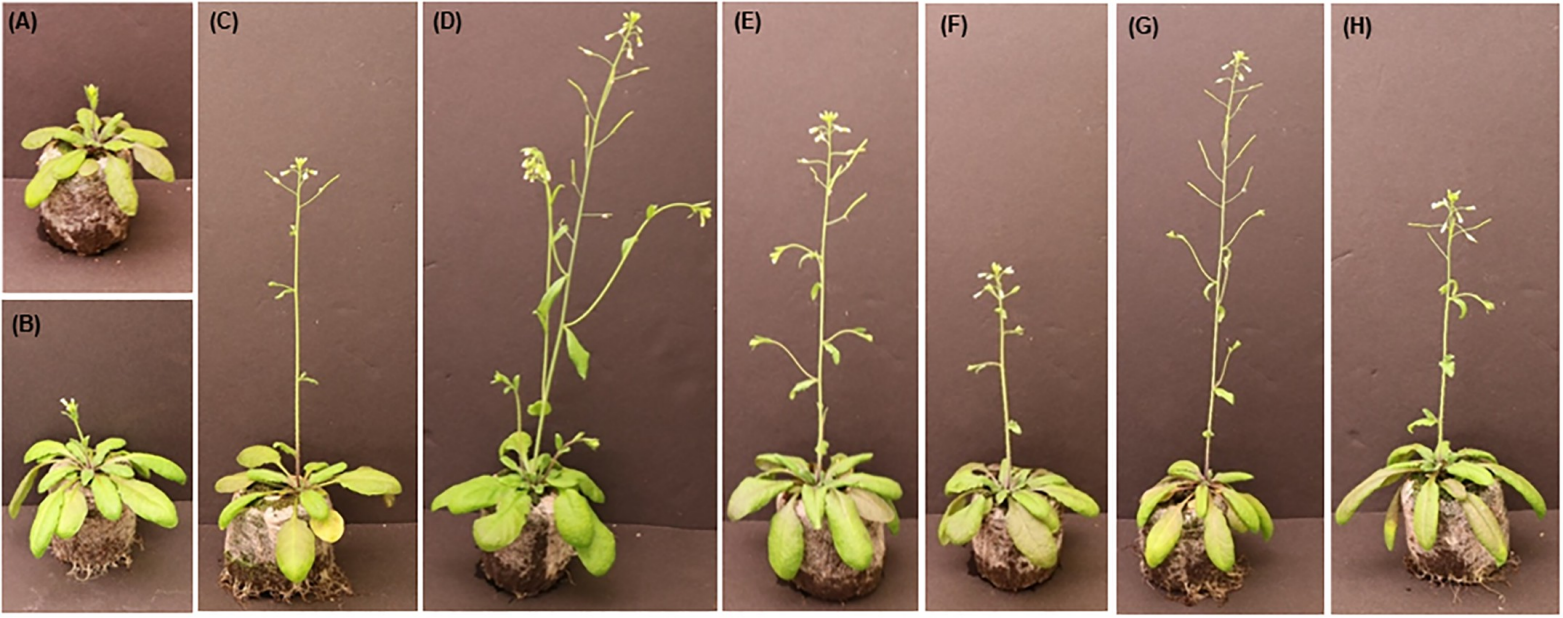
At 44 days wild type Col-0 had flowered (A and B). The *AtIPCS* over-expressing lines had also flowered at this time point, however all had bolted: (C) At1+; (D) At1++; (E) At2+; (F) At2++; (G) At3+; (H) At3++.

To examine the effects of these global alterations in gene expression on plant development, the phenotypes of *A*. *thaliana* over-expressing each of the *AtIPCS* isoforms (both levels) were analysed. All lines showed early flowering (4 days earlier than wild type Col-0) associated with the formation of bolts (Fig 6). This correlated with a slight (<2-fold log_2_) up-regulation of the florigen FT (see data deposited in GEO, Accession GSE129016), a well characterized systematic signal for plant transition from the vegetative to the reproductive (flowering) phase [41]. However, the mechanism underlying this phenotype remains unclear, and perhaps reflects the metabolic changes indicated in the analyses above.

Reflecting the broad negative regulatory effect *AtIPCS* over-expression has on biotic and abiotic stress responses in *Arabidposis* (transcriptomic data—Table 2 and Fig 4), the phenotypes of *Arabidopsis* At1++, At2++ and At3++ were observed under osmotic (abiotic) and pathogen (biotic) stress. Firstly, the over-expressing lines were analysed for tolerance to osmotic stress using the non-ionic osmolyte, mannitol (S4 Fig). In agreement with the down-regulation of genes involved in the abiotic stress response (Table 2; Fig 4), At1++, At2++ and At3++ over-expressing lines were all more susceptible to osmotic stress at high concentrations of mannitol (500mM).

**Fig 7:**
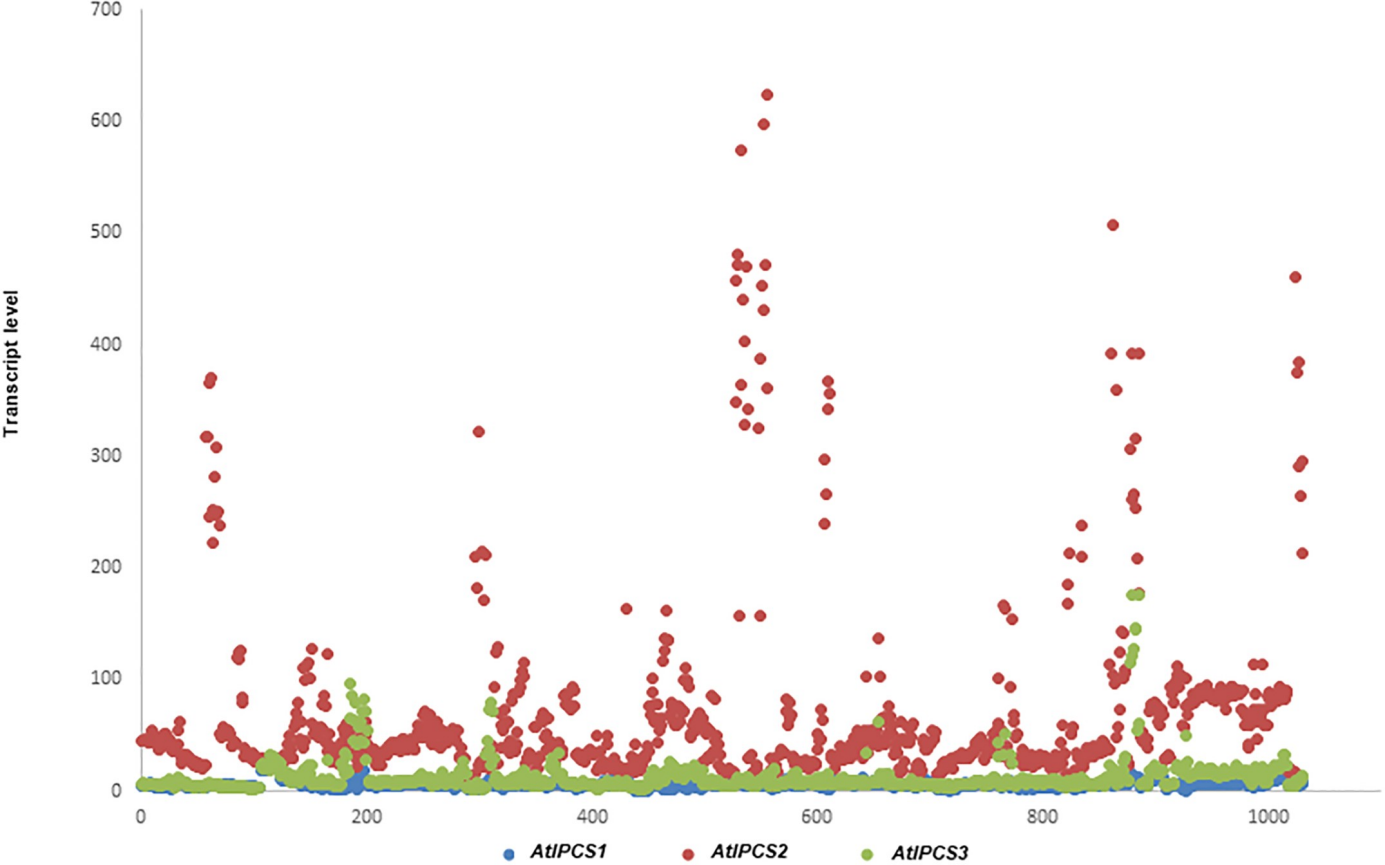
Predicted *AtIPCS1-3* expression in response to pathogens, pathogen effectors and chemical stimuli. x-axis: experiment number; y-axis: transcript level. Produced from RNA-Seq data using Genevestigator.

Subsequently, the phenotype of the pathogen response was assessed. Previously, a specific role for *At*IPSC2 in plant resistance to biotrophic pathogens (powdery mildew, *G*. *cichoracearum*) was proposed. A homozygote *At*IPSC2 T-insert mutant showed a reduction in fungal mass compared with a *G*. *cichoracearum* infected control, whereas resistance to the hemibiotrophic pathogen *Pseudomonas syringae* was unaffected [13]. The *At*IPSC2 T-insert mutant resistance to *G*. *cichoracearum* was associated with increased *PR1* [13], whereas as we demonstrated that *AtIPCS* over-expression reduced *PR1 (*and *PR2*) expression. This places *AtIPCS* at the centre of the biotic stress response. Therefore, to examine the potential role of *At*IPCS in the necrotophic pathogen response, *Arabidopsis* At1++, At2++ and At3++ were challenged with *Erwinia amylovora*. Interestingly, based on the spread of the pathogen across the surface of the leaves, At2++ and At3++, but not At1++, were less susceptible than the Col0 controls (S5 Fig). At first glance these results appear counter intuitive, the over-expression lines showing down-regulation of the biotic response transcriptome but phenotypically showing a rise in pathogen resistance. Therefore, these data were considered in light of the expression data available in Genevestigator for *At*IPCS1-3 (Fig 7). Response to various stimuli is observed for *AtIPCS2* with a fold increase of up to 600 compared to the highest 100 fold increase observed for *AtIPCS1* during developmental leaf senescence [42]. The highest increase in *AtIPCS2* transcript levels was found to be in response to plants treated with ozone to activate apoplastic reactive oxygen species (ROS) signalling, correlating with the increase in transcript associated with antioxidant flavonoid metabolism seen in At2++ (S3 Fig). *AtIPCS1* also showed an increase in the transcript, although 40-fold lower than *AtIPCS2* [43]. Notably, *AtIPCS1* and *2* transcript increases were also observed in ozone tolerant plants [44]. Other elicitors of *AtIPCS2* transcript increase include the fungi *Botryris cinereal* [45] and *Blumeria graminis* [46], the bacterium *P*. *syringae* [47], and bacterial flagellin protein [48]. Therefore, increased expression of *AtIPCS2* (and perhaps *AtIPCS*1 and 3) is part of the response to necrotropic, biotrophic and hemibiotropic pathogens. All this in a background of the suppression of the biotic stress response at the transcriptomic level.

## Discussion

Each of the 3 *IPCS* isoforms are differentially expressed in the tissues of *A*. *thaliana* [4], and in *Oryza sativa* (rice) *IPCS* expression in response to specific abiotic stimulus is tissue specific [15]. To further probe the downstream effects of IPCS, in this study we analysed the transcriptomic response to the over-expression of *AtIPCS1*, *2* and *3*.

Multiple genes responded both positively and negatively, and specifically, in response to elevated *AtIPCS1*, *2* and *3*. Analyses of genes up-regulated in response to *AtIPCS* over-expression showed most enrichment under GO term GO:0010876 (*lipid localization*), with levels showing strong correlation with increased expression of *AtIPCS1* and *3*, indicating a regulatory (rheostat) function (Table 5). Specifically, 2S SEED STORAGE PROTEIN 1, 2, 3 and 4 genes, LIPID TRANSFER PROTEIN gene (*LPT*; AT5G54740) and OLEOSIN 1, 2 and 4 genes (*OLEO1*, *2* and *4*) are up-regulated in response in *AtIPCS1*, and further up-regulated with increased expression. In addition, all apart from *OLEO2* responded similarly to *AtIPCS3* (Table 5), and all were up-regulated in response to *AtIPCS2* over-expression. Several other *LPT* genes also positively responded to *AtIPCS2* over-expression. *OLE* genes encode oleosins that prevent the abnormal fusion of oil bodies in seeds during imbibition and thereby protect the seeds from undergoing mechanical stress that would result in mortality [49]. Also upregulated were the 2S SEED STORAGE PROTEIN genes which act as nitrogen and sulphur reserves for seeds during germination [50]. Both were particularly influenced by the expression levels of *AtIPCS1* and 3 (Table 5) suggesting these isoforms have a role in seed development. A small number of genes were enriched under GO term GO:000979 (*post-embryonic development*) and up-regulated in response to *AtIPCS1* and *3* (S4 and S6 Tables). An shown in S7 Table, the effects seen with *AtIPCS1* and 3 were again magnified on increased expression. Notably, in addition to those genes already discussed, up-regulated CRUCIFERIN 2 and 3 genes (*CRU2* and *3*) are seed storage proteins expressed during the later stages of embryogenesis [39] with CRU3 having a role in protein and oil storage [40]. From these data, it appears that *AtIPCS* is involved in regulating protein and lipid storage in seeds. This potential role is further supported by the observation that 1-CYSTEIN PEROXIREDOXIN 1 (*AtPER1*) and EXTENSIN PROLINE-RICH 1 (*AtEPR1*) are also up-regulated in response to *AtIPCS*. Both are expressed in the embryo and in developing seeds, providing protection from ROS during seed desiccation [51, 52]. EMBRYOGENIC CELL PROTEIN 31 gene (*AtECP31*) and CALEOSIN PROTEIN gene 1 (*AtCOL1*), both expressed in the later stages of seed development, are important for seed viability and desiccation tolerance [53, 54]. Therefore, *At*IPCS may be important in the protection, viability and therefore the germination of seeds. The mechanisms behind this are not known, however the function of these enzymes in regulating ceramide and phytoceramide levels point towards multiple roles in the signal transduction networks underlying development [5].

The next most enriched genes up-regulated in response to *AtIPCS* over-expression were under GO term GO:0015979 (*photosynthesis*). The importance of this in relation to IPCS functionality is unclear. However, the most compelling change in transcript levels was seen under the influence of *AtIPCS2* over-expression; whilst *AtIPCS3* appeared to function as a rheostat (Table 4) with increased expression inducing the up-regulation of all 6 genes influenced (≥2 log_2_). Notably this isoform is least expressed in rosette and cauline leaves of *A*. *thaliana* [4] perhaps indicating a role of up-regulation in photosynthetic regulation. However, the mechanism behind this possible function is unclear.

To further visualise the possible effects of *At*IPCS over-expression, and given the scale and scope of its influence on the transcriptome, the *AtIPCS2* higher level over-expressor (At2++) data was analysed using MapMan (S3 Fig). The expression of many genes associated with metabolism were positively influenced, particularly those associated light reactions and antioxidative flavonoids. Further analyses showed up-regulated genes to be enriched under photorespiration, the Calvin cycle and light reactions (Fig 5), perhaps positively influencing energy production and the rate of growth. Interestingly, all *At*IPCS over-expressing lines showed bolts (Fig 6) perhaps indicating accelerated growth, however this requires further analysis.

Flavonoid metabolism was also up-regulated in At2++ *Arabidopsis* at the transcriptome level, and these antioxidant molecules are synthesized to protect plant tissues from ROS produced in response to abiotic and biotic stress [38]. Therefore, over-expression of *At*IPCS2 may also have a protective role in plant defense, a stress response. Interestingly, and perhaps correlating with this, on over-expression of each of the isoforms down-regulated genes were significantly enriched under GO term GO:0006950 (*response to stress*) (S1–S3 Tables). These included Pathogenesis-Related (*PR*) genes, *PR1* and 2 were particularly effected and transcript levels were reduced ≥2 log_2_ (Table 2), this effect was magnified on increased expression of *AtIPCS1* suggesting that this isoform may have a regulatory role in the stress response. (Table 2). Systematic Acquired Resistance (SAR) is characterised by the increased expression of the *PR* genes which are induced in response to elevated endogenous growth hormones such as salicylic acid (SA) and ethylene (ET), the levels of which increase in response to infection [55]. *A*. *thaliana* manipulated to express elevated levels of *PR1*, *2* and *5* are resistant to the oomycete obligate biotroph *Hyaloperonospora parasitica* and the bacterium biotroph *Pseudomonas syringae pv*. *Maculicola* [56]. Furthermore, *PR* genes are also induced by environmental stress such as cold and light [57]; *PR1*, *PR2* and *PR5* were induced in cold treated and drought stressed *A*. *thaliana* [58, 59].

Plant Defensin genes, *PDF1*.*2B* and an ET- AND JA-RESPONSIVE PLANT DEFENSIN, are similarly repressed in response to over-expression of *AtIPCS1*, *2* and *3*, and again the effect was magnified on increased expression of (rheostatic) *AtIPCS1* (Table 2). Like the *PR* genes, *PDF* genes are markers of SAR induced by endogenous growth hormones in response to biotic and abiotic stress [55]. Wound associated TYROSINE AMINO TRANSFERASE 3 (TAT3) was also induced in *A*. *thaliana* in response to an endogenous growth hormone (JA) [60], and was down-regulated in response to all isoforms but with a magnified effect on increased expression of *AtIPCS1* (Table 2). LATE UP-REGULATED IN RESPONSE TO *HYALOPERONOSPORA PARASITICA* (*LURP1)* was down-regulated in response to over-expression of all isoforms and, as the name suggests, has been shown to be needed for basal resistance to the oomycete *Hyaloperonospora parasitic* [61]. More specifically PHOSPHOLIPASE 2A (*PLA2A*) expression is negatively regulated only by over-expression of *AtIPCS2*. An orthologue of this enzyme is induced in mosaic virus infected tobacco leaves independently of the growth hormone JA [62] (Table 2).

Similarly, in relation to abiotic stress response, MYB112, which is induced in salt stressed plants [63], responded specifically and negatively to *AtIPC2* over-expression. CIRCADIAN CLOCK ASSOCIATED 1 (*CCA1*) and GIGANTEA (*GI*), which are part of the photoperiodic control of flowering [64, 65], are specifically negatively regulated by over-expression of *AtIPCS2* and *3*, and *AtIPCS2* respectively (Table 2). Notably, in support of these effects, the *AtIPCS* over-expressing lines displayed an early flowering phenotype.

These data indicating the role of *AtIPCS* over-expression in the suppression of biotic and, to a lesser extent, abiotic stress responses, are supported by the MapMan analyses (Fig 4). At a phenotypic level, the transcriptomic findings correlate with a decreased tolerance for osmotic (abiotic) stress, albeit only at high concentrations of the non-ionic osmolyte, mannitol (S5 Fig). However, the relative resistance of the *At*IPCS2 and 3 over-expressor lines (At2++ and At3++) to challenge with the necrotroph *Erwinia amylovora* (S4 Fig) is difficult to reconcile with the transcriptomic data showing down-regulation of the pathogen response. However, Genevestigator analyses (Fig 7) indicated that increased expression of *AtIPCS2* (and perhaps *AtIPCS*1 and 3) is part of the response to necrotropic, biotrophic and hemibiotropic pathogens. The mode of this in biotic stress response is unclear, however Genevestigator showed that the *AtIPCS2* transcript level positively correlated with ROS signalling, as could the indicated increase in antioxidant flavonoid metabolism noted in this work (S3 Fig). Clearly this warrants further investigation, however it notable that the response to *E*. *amylovora*, and other pathogens, in *Arabidopsis* includes ROS [66].

Together these data suggest some specificity in the influence of each *AtIPCS* isoform and that *AtIPCS1* expression may act as a rheostat of SAR and the response to biotic and abiotic stress. Furthermore, the observation that *AtIPCS* expression negatively influences both growth hormone dependent (e.g. *PR*) and independent responses (*PL2A*) indicated its role in a wide variety of defence networks perhaps reflecting the role of phytoceramide as an indiscriminate pro-apoptotic signal [13].

## Conclusion

Transcriptomic analyses of *A*. *thaliana* indicated that *AtIPCS1-3* over-expression positively correlated with the expression of genes encoding storage proteins essential for normal seed development (S7 Table). As such, the enzyme may be crucial for seed survival, maturation and germination. Furthermore, these data also indicated that *AtI*PCS acts as a negative regulator of the plant defense response to pathogens and abiotic stress (Tables 2–4), a process associated with PCD. Importantly, these findings were also corroborated by data available from Genevestigator (Fig 7) and phenotypic observations (S4 and S5 Figs).

The negative association of biotic and abiotic stress responses to *AtI*PCS expression indicates the potential to engineer tolerance in crop plants.

## Supporting information

S1 Table GO enrichment of genes down-regulated in response to the constitutive over-expression of *AtIPCS*. (TIF)

S2 Table GO enrichment of genes down-regulated in response to the constitutive over-expression of *AtIPCS2*. (TIF)

S3 Table GO enrichment of genes down-regulated in response to the constitutive over-expression of *AtIPCS3*. (TIF)

S4 Table GO enrichment of genes up-regulated in response to the constitutive over-expression of *AtIPCS1*. (TIF)

S5 Table GO enrichment of genes up-regulated in response to the constitutive over-expression of *AtIPCS2*. (TIF)

S6 Table GO enrichment of genes up-regulated in response to the constitutive over-expression of *AtIPCS3*. (TIF)

S7 Table Genes showing a positive correlation with *AtIPCS* expression under GO term GO: 0009791 (*post-embryonic development*). At1-3+ over-expressing *AtIPCS1-3*; At1-3++ higher level expressers of *AtIPCS1-3*. Log_2_ change ≥1 shown, ≥2 in bold.
(TIF)

S1 Fig Relative quantitation of mRNA levels of (A) *At*IPCS1 (B) *At*IPCS2 (C) *At*IPCS3 in over-expressor transgenic lines compared to Col0 standardised to equate to a value of 1; qPCR was performed to measure mRNA levels in 10-day old seedlings. Relative quantitation was done after normalization using PEX4 levels; relative quantitation value is the mean of three biological replicates with standard error.
(TIF)

S2 Fig Metabolisim overview generated in MapMan for genes down-regulated in At2++ transgenic line. Log_2_ fold changes in gene expression are indicated by the colour scale, with the distribution of genes in different pathways and expression levels shown. Abbreviations: carbohydrates (CHO), tricarboxylic acid (TCA) cycle, oxidative pentose phosphate (OPP) pathway, sulphur containing glucosinates synthesis (S-misc), nitrogen containing glucosinate synthesis (N-misc).
(TIF)

S3 Fig Metabolisim overview generated in MapMan for genes up-regulated in At2++ transgenic line. Log_2_ fold changes in gene expression are indicated by the colour scale, with the distribution of genes in different pathways and expression levels shown. Abbreviations: carbohydrates (CHO), tricarboxylic acid (TCA) cycle, oxidative pentose phosphat pathway, sulphur containing glucosinates synthesis (S-misc), nitrogen containing glucosinate synthesis (N-misc).
(TIF)

S4 Fig 8 day *Arabidopsis thaliana* seedlings treated with the non-ionic osmolyte mannitol for a further 6 days. Col0 and overexpressing lines At1++, At2++ and At3++. Mannitol concentrations in mM. At the highest concentration (500mM) chlorosis in the over-expressing lines, but not Col0, was apparent.
(TIF)

S5 Fig *Arabidopsis thaliana* leaves challenged with *Erwinia amylovora* 3 dpi. (A) Col0 (B) At1++ (C) At2++ (D) At3++ (E) Plot of ratio of area infected by *Erwinia amylovora* to uninfected area for Col0 and over-expression lines. *At*IPCS2 and 3 over-expressors are less susceptible to the pathogen compared to Col0 and *At*IPCS1 over-expressor, based on the spread of the pathogen across the surface of the leaf. The pathogen has spread to occupy a large area of Col0 and *At*IPCS1 over-expressor leaves, compared to a less aggressive spread seen on the leaves of *At*IPCS2 and 3 over-expressors. In addition, a distinctive yellow colour is observed at the outer boundaries of the area occupied by the pathogen, indicating a measured response that favours plant survival. These observations may be linked to the role of *At*IPCS as a negative regulator of the plant response to biotic stress.
(TIF)
