# Supplementary figures and images for: Expression levels of inositol phosphorylceramide synthase modulate plant responses to biotic and abiotic stress in Arabidopsis thaliana
Source: PLoS One. 2019 May 23;14(5):e0217087. doi: 10.1371/journal.pone.0217087 (PMC6532887; doi:10.1371/journal.pone.0217087)

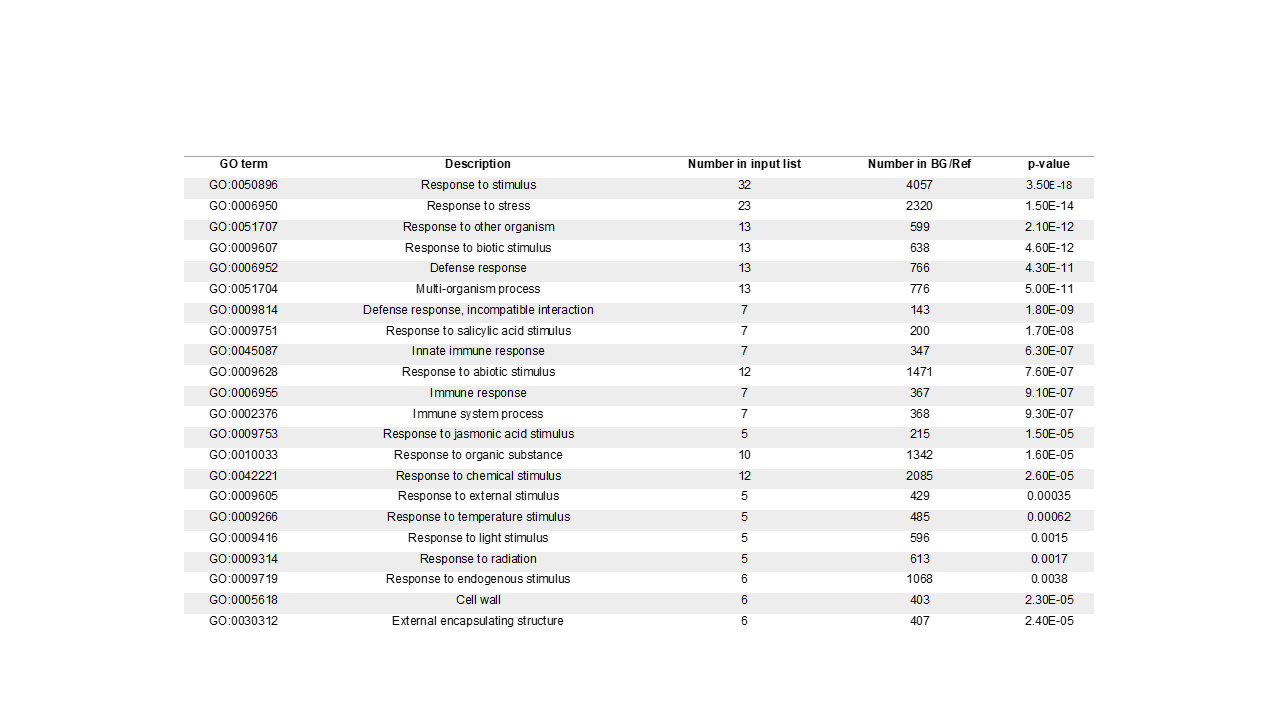

Supplement: S1 Table — (TIF) [file pone.0217087.s001.tif]

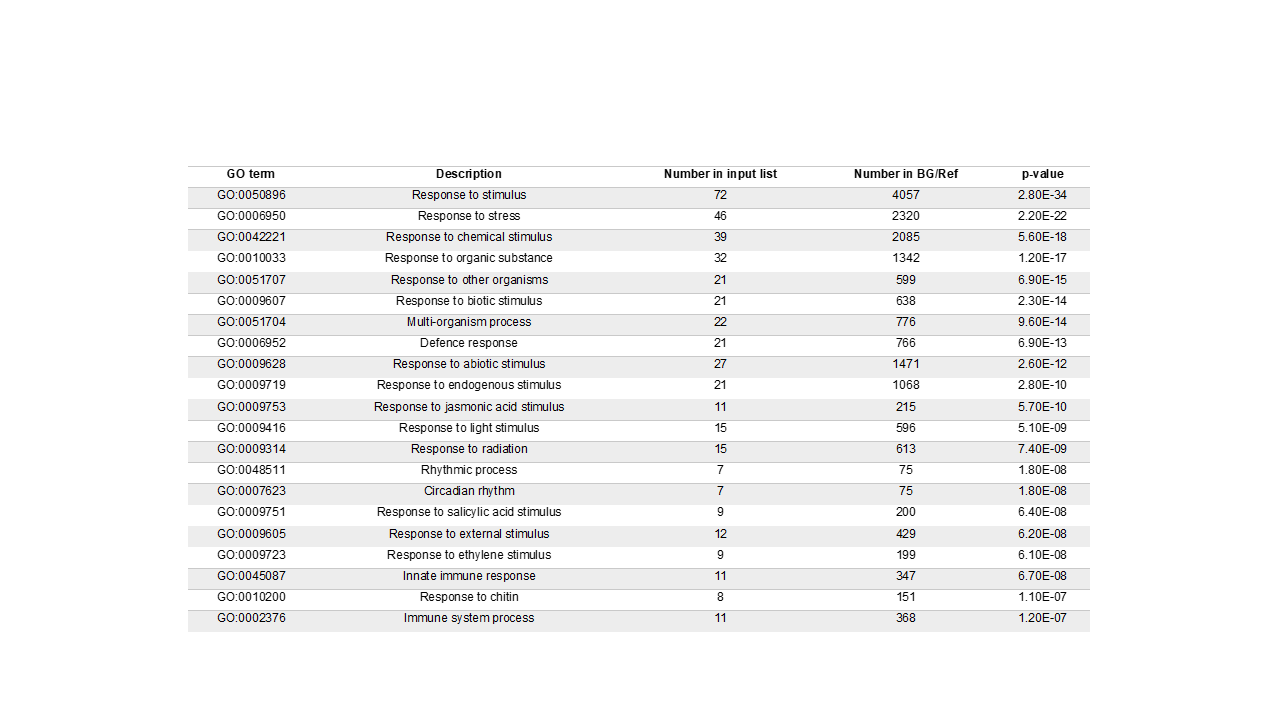

Supplement: S2 Table — (TIF) [file pone.0217087.s002.tif]

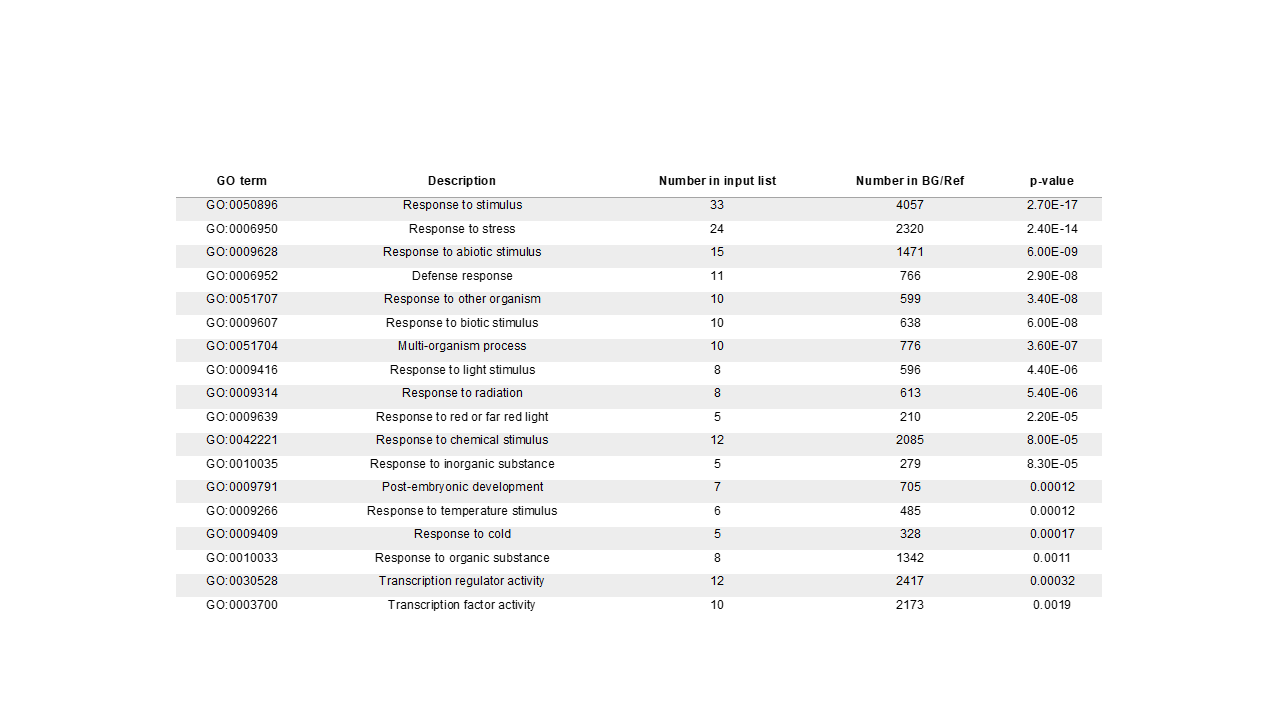

Supplement: S3 Table — (TIF) [file pone.0217087.s003.tif]

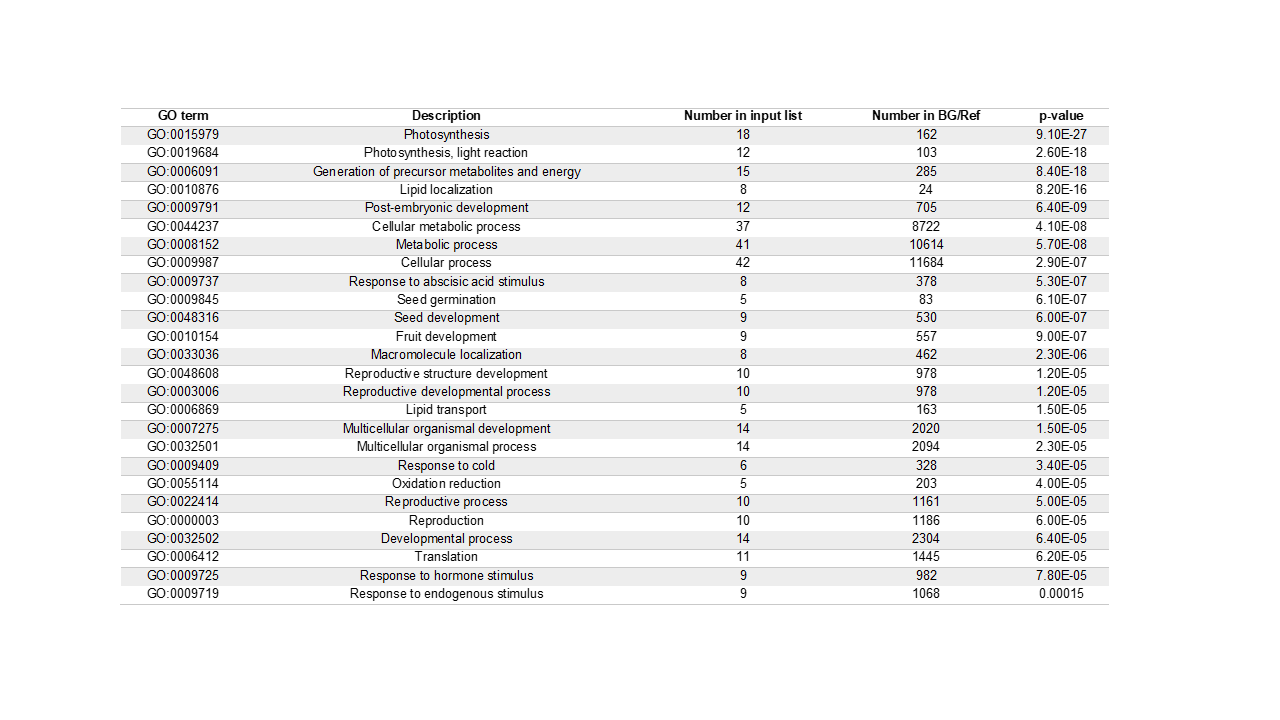

Supplement: S4 Table — (TIF) [file pone.0217087.s004.tif]

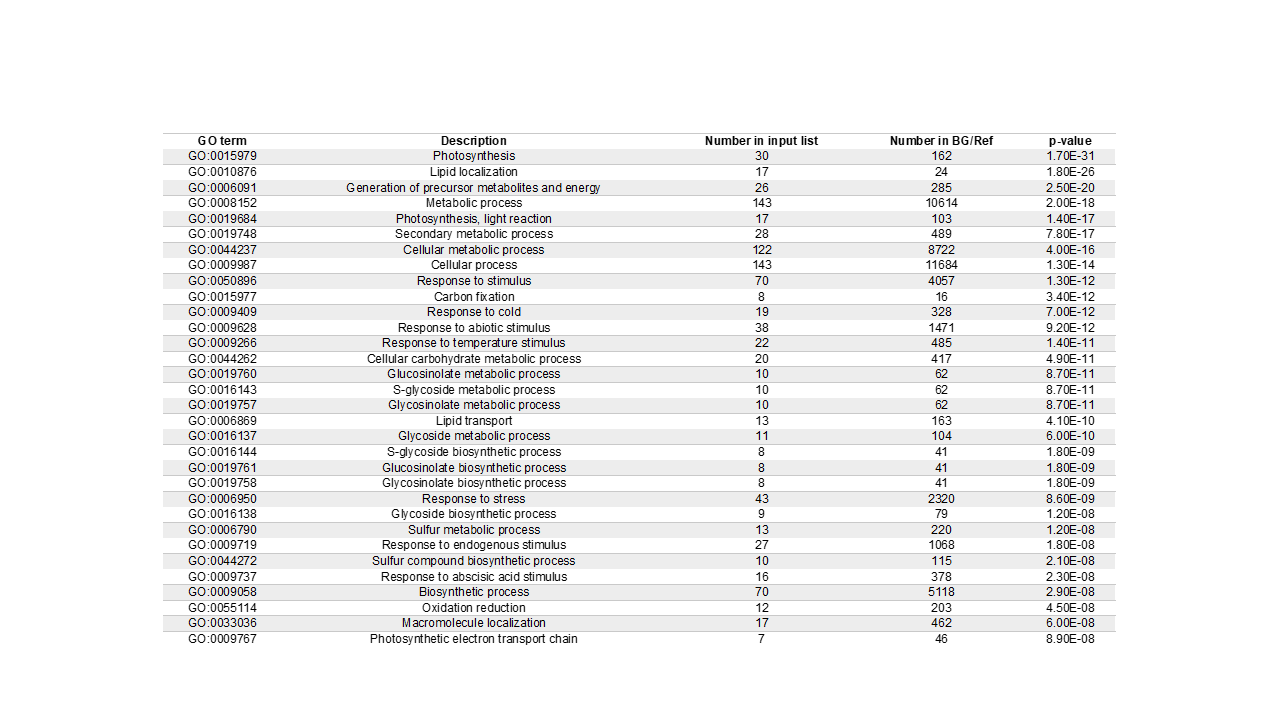

Supplement: S5 Table — (TIF) [file pone.0217087.s005.tif]

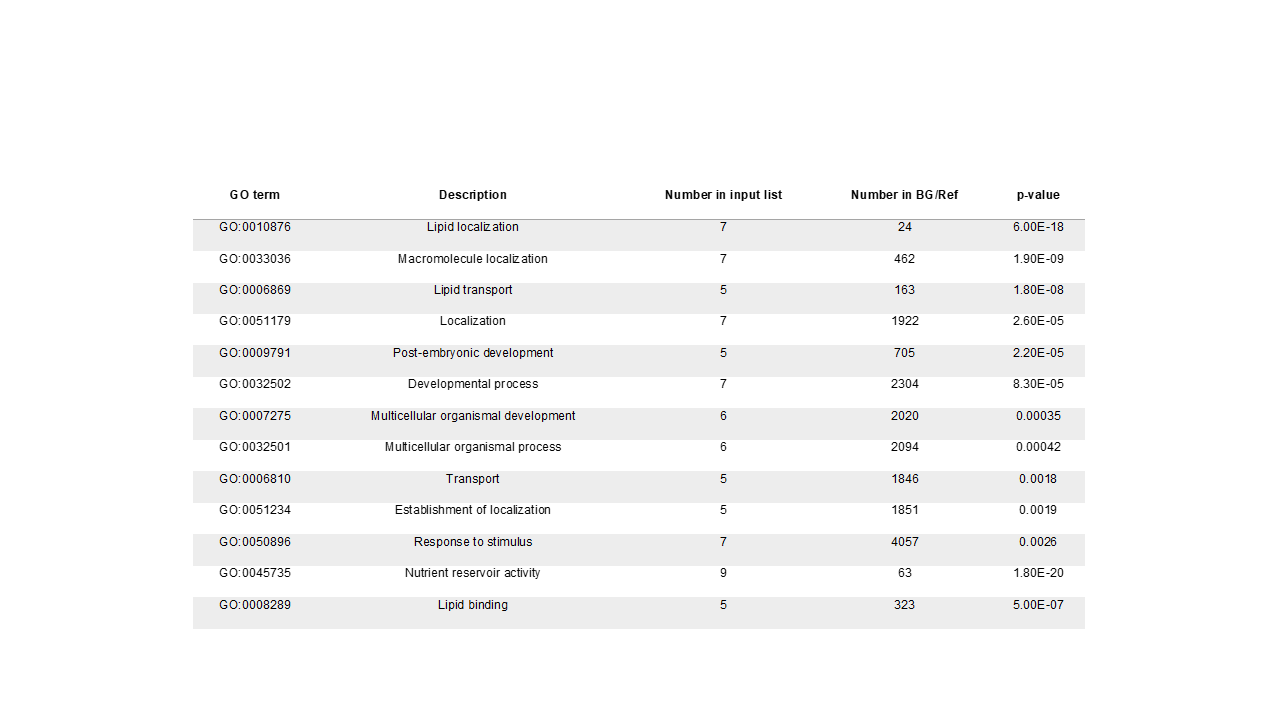

Supplement: S6 Table — (TIF) [file pone.0217087.s006.tif]

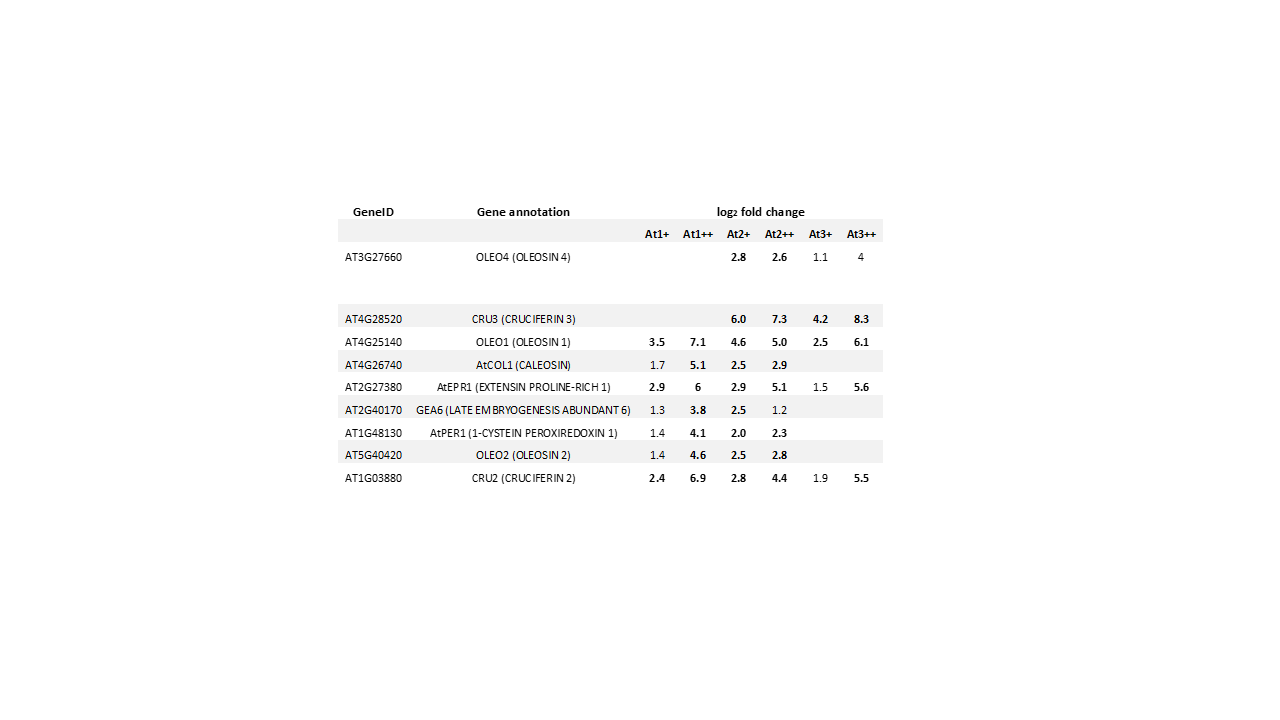

Supplement: S7 Table — At1-3+ over-expressing AtIPCS1-3; At1-3++ higher level expressers of AtIPCS1-3. Log2 change ≥1 shown, ≥2 in bold. (TIF) [file pone.0217087.s007.tif]

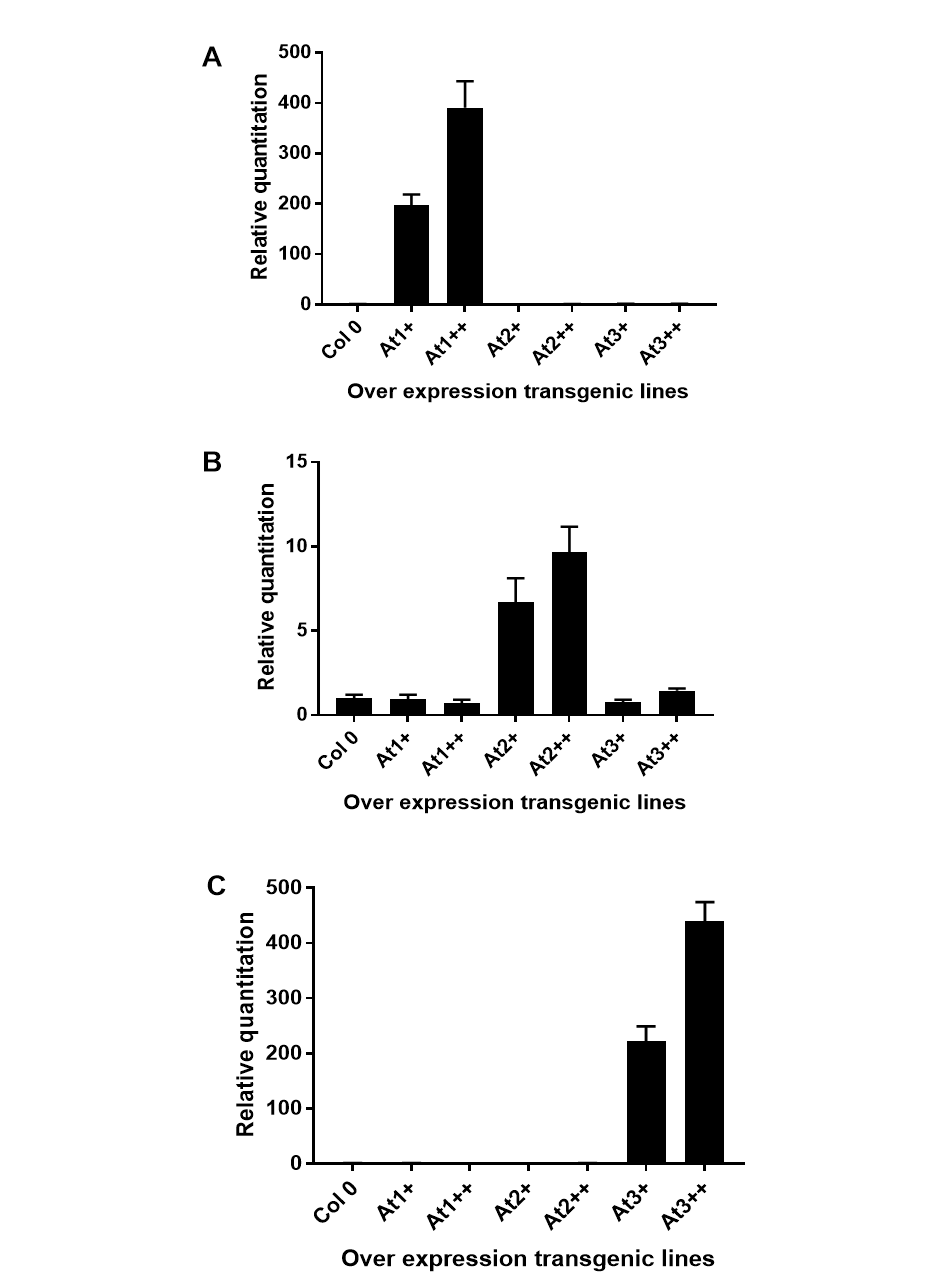

Supplement: S1 Fig — Relative quantitation was done after normalization using PEX4 levels; relative quantitation value is the mean of three biological replicates with standard error. (TIF) [file pone.0217087.s008.tif]

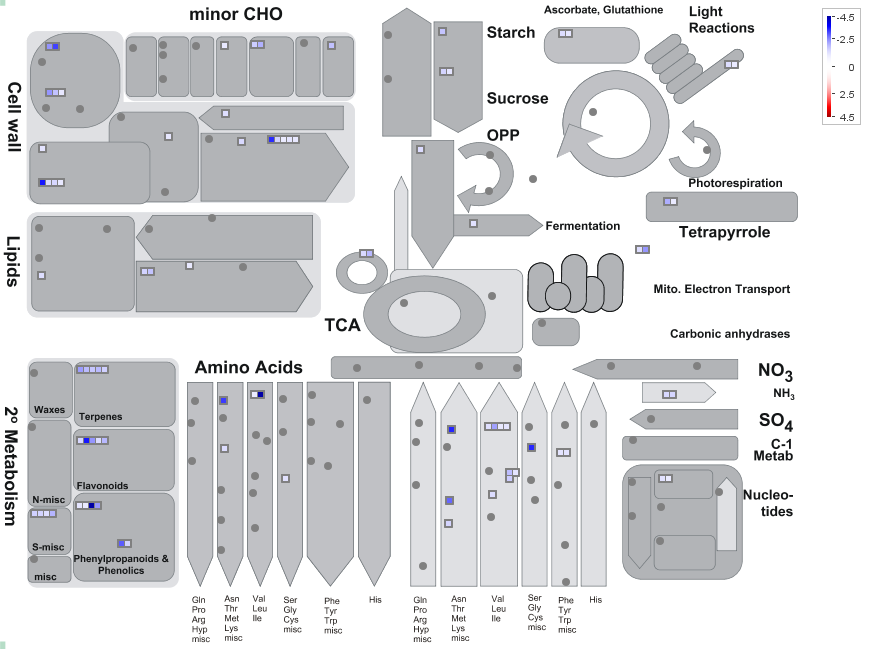

Supplement: S2 Fig — Log2 fold changes in gene expression are indicated by the colour scale, with the distribution of genes in different pathways and expression levels shown. Abbreviations: carbohydrates (CHO), tricarboxylic acid (TCA) cycle, oxidative pentose phosphate (OPP) pathway, sulphur containing glucosinates synthesis (S-misc), nitrogen containing glucosinate synthesis (N-misc). (TIF) [file pone.0217087.s009.tif]

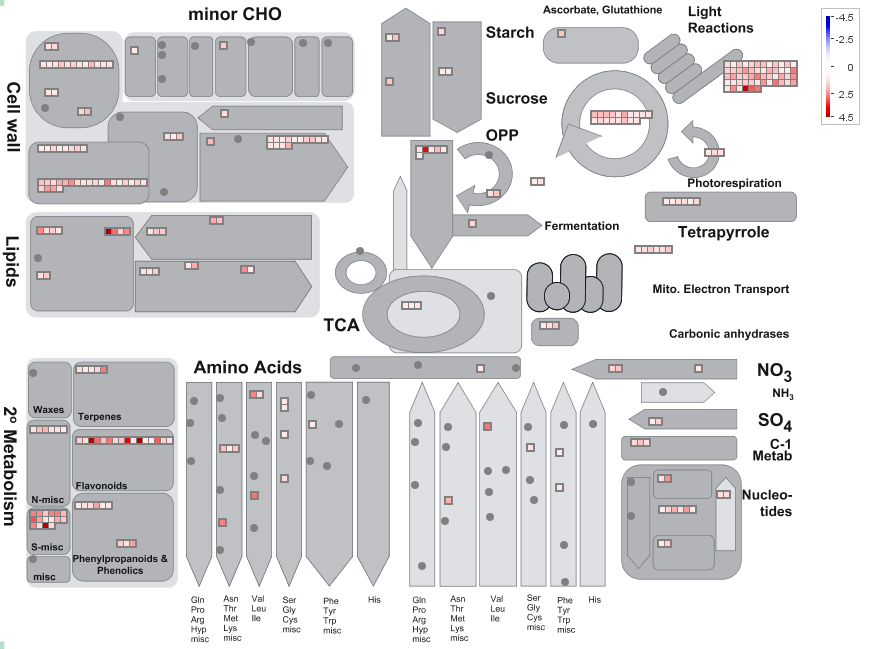

Supplement: S3 Fig — Log2 fold changes in gene expression are indicated by the colour scale, with the distribution of genes in different pathways and expression levels shown. Abbreviations: carbohydrates (CHO), tricarboxylic acid (TCA) cycle, oxidative pentose phosphat pathway, sulphur containing glucosinates synthesis (S-misc), nitrogen containing glucosinate synthesis (N-misc). (TIF) [file pone.0217087.s010.tif]

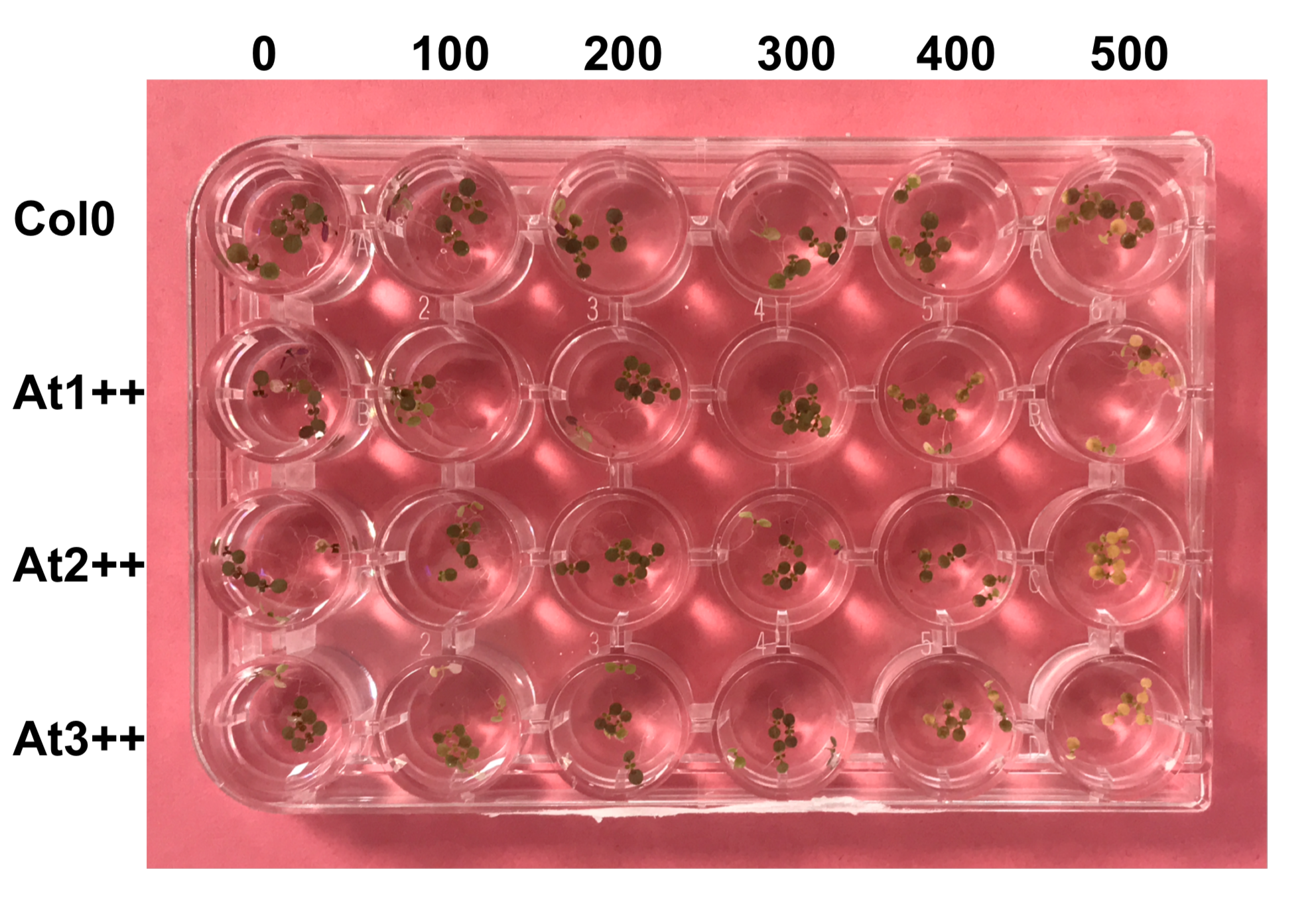

Supplement: S4 Fig — Col0 and overexpressing lines At1++, At2++ and At3++. Mannitol concentrations in mM. At the highest concentration (500mM) chlorosis in the over-expressing lines, but not Col0, was apparent. (TIF) [file pone.0217087.s011.tif]

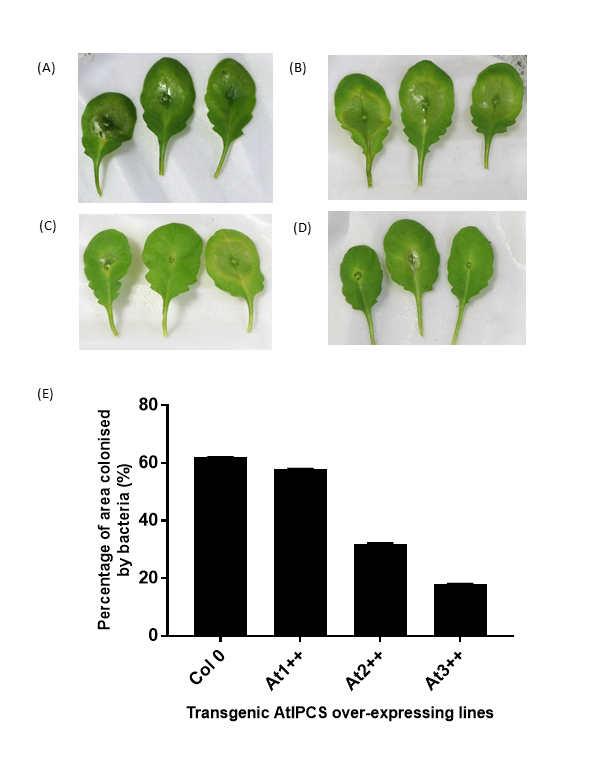

Supplement: S5 Fig — (A) Col0 (B) At1++ (C) At2++ (D) At3++ (E) Plot of ratio of area infected by Erwinia amylovora to uninfected area for Col0 and over-expression lines. AtIPCS2 and 3 over-expressors are less susceptible to the pathogen compared to Col0 and AtIPCS1 over-expressor, based on the spread of the pathogen across the surface of the leaf. The pathogen has spread to occupy a large area of Col0 and AtIPCS1 over-expressor leaves, compared to a less aggressive spread seen on the leaves of AtIPCS2 and 3 over-expressors. In addition, a distinctive yellow colour is observed at the outer boundaries of the area occupied by the pathogen, indicating a measured response that favours plant survival. These observations may be linked to the role of AtIPCS as a negative regulator of the plant response to biotic stress. (TIF) [file pone.0217087.s012.tif]
